# The geroprotectors trametinib and rapamycin combine additively to extend mouse healthspan and lifespan

**DOI:** 10.1038/s43587-025-00876-4

**Published:** 2025-05-28

**Authors:** Lisonia Gkioni, Tobias Nespital, Maarouf Baghdadi, Carolina Monzó, Jitin Bali, Taim Nassr, Anna Lena Cremer, Andreas Beyer, Joris Deelen, Heiko Backes, Sebastian Grönke, Linda Partridge

**Affiliations:** 1 Department Biological Mechanisms of Ageing, Max Planck Institute for Biology of Ageing, Cologne, Germany; 2 Institute for Integrative Systems Biology, Spanish National Research Council, Paterna, Spain; 3 Cellular Networks and Systems Biology Group, CECAD Research Centre, Cologne, Germany; 4 Multimodal Imaging of Brain Metabolism Group, Max Planck Institute for Metabolism Research, Cologne, Germany; 5 Institute of Healthy Ageing and GEE, University College London, London, UK

**Keywords:** Ageing, Mouse

## Abstract

Suppression of the insulin–IGF–mTORC1–Ras network ameliorates aging in animals. Many drugs have targets in the network because of its roles in cancer and metabolic disease and are candidates for repurposing as geroprotectors. Rapamycin, an established geroprotective drug, blocks mTORC1 signaling, and trametinib inhibits the Ras–MEK–ERK pathway. In this study, we assessed survival and health of male and female mice treated with trametinib, rapamycin or their combination. We show here that trametinib treatment extended lifespan in both sexes and that its combination with rapamycin was additive. Combination treatment reduced liver tumors in both sexes and spleen tumors in male mice, blocked the age-related increase in brain glucose uptake and strongly reduced inflammation in brain, kidney, spleen and muscle and circulating levels of pro-inflammatory cytokines. We conclude that trametinib is a geroprotector in mice and that its combination with rapamycin is more effective than either drug alone, making the combination a candidate for repurposing as a gerotherapy in humans.

## Abstract (web summary)

Gkioni et al. show that trametinib, which targets the Ras–Mek–Erk pathway, extends lifespan in male and female mice and that co-treatment with the known geroprotector rapamycin additively extends lifespan and healthspan, reducing inflammation and tumor growth.

## Main

The insulin–IGF–mTORC1–Ras nutrient-sensing network is highly conserved in evolution and is implicated in the etiology of many age-related diseases^1,2^. Genetic inhibition of multiple nodes within the network can improve health during aging and extend lifespan in laboratory model organisms^3^. Growing evidence indicates a pro-aging role of the network in humans^1,4^. There is, therefore, potential to repurpose existing drugs with targets in this signaling network as geroprotectors to improve human health during aging^5^.

Inhibition of mTORC1 by rapamycin (sirolimus), a US Food and Drug Administration (FDA)-approved drug used mainly for immunosuppression after organ transplantation^6^, robustly extends lifespan in multiple model organisms^7,8^, including mice, where rapamycin administration later in life at 600 days of age increases median and maximal lifespan in both sexes^9^. Rapamycin also ameliorates aging-related morbidities, including cardiac dysfunction^10^ and impaired immune responses^11^. Administration of mTOR inhibitors to older human subjects led to enhanced immune response to influenza vaccine and reduced infection rate, with briefer and lower dosing than used clinically and minimal side effects^12^. Some of the geroprotective effects of rapamycin may, therefore, be conserved in humans.

Reduced signaling through the phosphatidylinositol 3-kinase (PI3K) node of the nutrient-sensing network can extend lifespan in *Caenorhabditis elegans* and *Drosophila*^13,14^ and has been viewed as the primary route mediating the anti-aging effects of reduced upstream insulin/IGF signaling. However, Ras signaling plays a role in aging in yeast^15^, whereas, in *Drosophila*, the Ras–MEK–ERK pathway is as important a mediator as the PI3K pathway of the upstream effects of insulin/IGF signaling on lifespan^16^. Indirect inhibition of Ras in mice is associated with increased lifespan and enhanced motor function in old age^17^. Inhibition of the Ras pathway may, therefore, have an evolutionarily conserved, geroprotective effect.

Ras hyperactivation is highly oncogenic, with a third of human cancers presenting with a Ras mutation^12^. There has, therefore, been an intense search for small-molecule inhibitors of the Ras pathway. Trametinib (also known as Mekinist), an FDA-approved drug for treatment of melanomas, is a highly specific inhibitor of MEKs^18^. Oral administration of trametinib increases *Drosophila* lifespan, even when started later in life^16^. To examine whether the geroprotective effect of trametinib is conserved in mice, we orally dosed female and male mice and assessed their aging phenotypes.

There is extensive crosstalk among the branches of the insulin–IGF–mTORC1–Ras network. Simultaneous inhibition of different nodes within the network, by combined drug treatments, could, therefore, be more effective than suppression of single nodes, by prevention of compensatory responses. Combinatorial drug treatments could also inhibit specific targets more strongly or induce a wider range of protective effects, because different nodes in the network have many non-overlapping targets. Indeed, joint inhibition of mTOR, MEK and glycogen synthase kinase-3 (GSK-3) by rapamycin, trametinib and lithium, respectively, resulted in additive increases in *Drosophila* lifespan, both with both double and triple treatment combinations, the latter inducing a 48% median lifespan extension^19^. Such combinatorial drug treatment could exert similar benefits in mammalian aging.

In the present study, we investigated whether administration of trametinib alone or in combination with rapamycin can extend lifespan and improve health at old age in mice. We orally treated male and female mice with trametinib or rapamycin or with both drugs at the same doses as in the single-drug treatments. We assessed their survival, fitness, brain metabolism and organismal health. Administration of trametinib or rapamycin alone significantly increased male and female lifespan, whereas the combination produced an additive increase in both sexes. Additionally, combined treatment significantly reduced liver tumors in both sexes and spleen tumors in male mice and alleviated the age-related increased glucose uptake in the brain. Combination treatment also markedly reduced age-related inflammation in brain, kidney, spleen and muscle, with reduced levels of circulating pro-inflammatory cytokines. Trametinib also is, thus, geroprotective in mice, and its combination with rapamycin is even more so.

## Results

### Dose range for oral administration of trametinib in mice

**Fig. 1:**
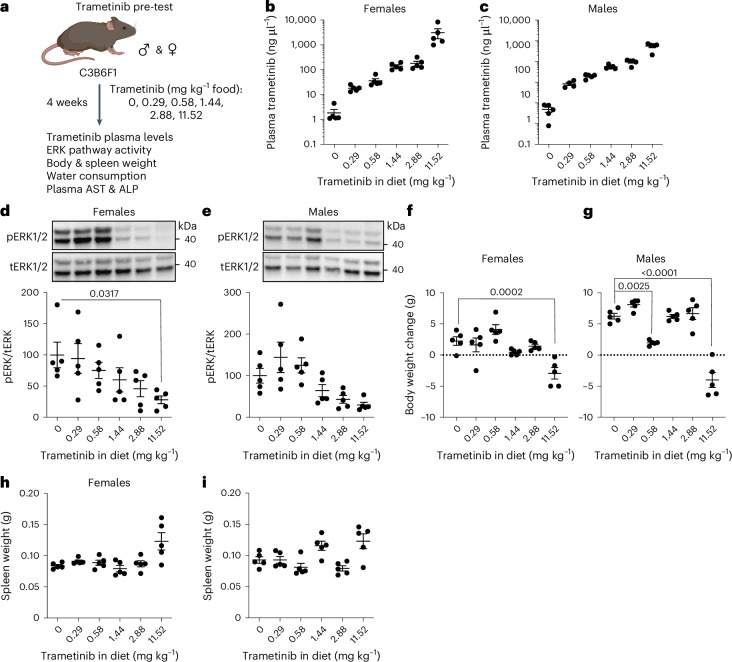
Doses of trametinib that inhibit Ras/MEK/Erk signaling in vivo in mice without detrimental side effects. **a**, Schematic outline of the trametinib pre-test experiment. Four-month-old C3B6 F1 hybrid mice were used for all experiments. **b**,**c**, Plasma concentration of trametinib in female (**b**) and male (**c**) mice (*n* = 5 plasma samples per treatment and sex, except *n* = 4 for male 0.29 mg kg^−1^) fed for 4 weeks with food containing 0, 0.29, 0.58, 1.44, 2.88 or 11.52 mg kg^−1^ trametinib. (One-way ANOVA with Bonferoniʼs post hoc test adjusted *P* values: female mice: 0 versus 11.52 *P* = 0.0073, 0.29 versus 11.52 *P* = 0.0077, 0.58 versus 11.52 *P* = 0.0082, 1.44 versus 11.52 *P* = 0.0116, 2.88 versus 11.52 *P* = 0.01; male mice: 0 versus 11.52 *P* < 0.0001, 0.29 versus 11.52 *P* < 0.0001, 0.58 versus 11.52 *P* < 0.0001, 1.44 versus 11.52 *P* < 0.0001, 2.88 versus 11.52 *P* < 0.0001). Female mice had significantly higher trametinib plasma levels than male mice across all doses, with a significant interaction of sex and drug (*P* = 0.0111, sex × drug, mixed model). **d**,**e**, Ras–Mek–Erk pathway activity in liver after 4 weeks of trametinib treatment, by western blot analysis of Erk1/2 phosphorylation. Representative western blot (upper panel) and corresponding quantification of female (**d**) and male (**e**) liver samples (*n* = 5 per treatment for both sexes). Trametinib concentrations of 1.44 mg kg^−1^ and higher inhibited Erk1/2 phosphorylation in liver of both sexes. **f**,**g**, Change in body weight upon 4 weeks of trametinib treatment in female (**f**) and male (**g**) mice. 11.52 mg kg^−1^ trametinib led to a significant reduction in body weight in both sexes compared to control animals (0 mg kg^−1^) (*n* = 5 animals per treatment). **h**,**i**, Wet spleen weight of female (**h**) and male (**i**) mice after 4 weeks of trametinib treatment. Spleen weight showed a tendency to increase with 11.52 mg kg^−1^ trametinib treatment. One-way ANOVA with Tukeyʼs post hoc test. Data are presented as mean ± s.e.m. Source data

**Extended Data Fig. 1:**
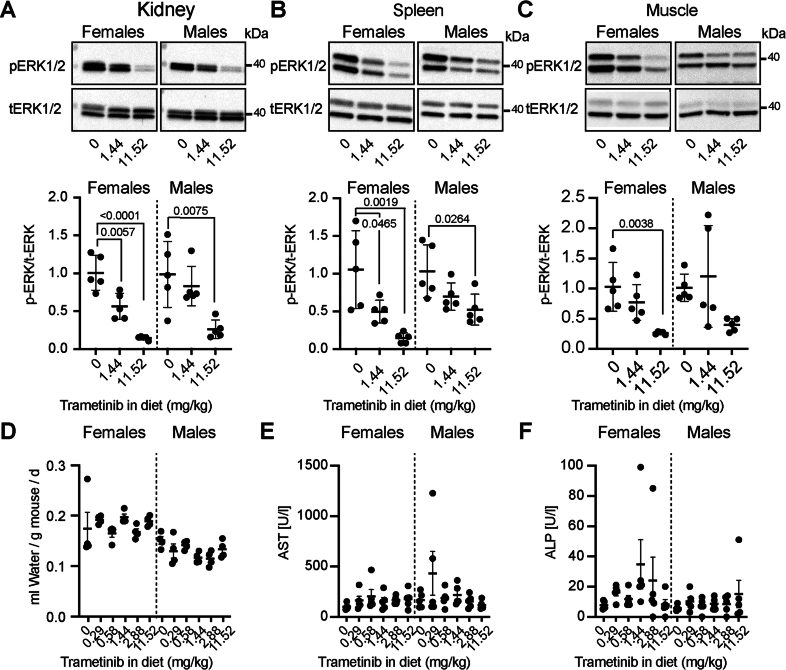
Short-term trametinib treatment reduces Ras/Mek/Erk pathway activity but has no effect on water consumption or biomarkers of liver health. **a**–**c** Western blot analysis of Erk1/2 phosphorylation as a proxy for Ras/Mek/Erk pathway activity in **a** kidney, **b** spleen and **c** muscle of females and males after 4 weeks of treatment with 0, 1.44 and 11.52 mg/kg trametinib. Representative Western blot (upper panel) and corresponding quantification (lower panel) (n = 5 tested organs of individual animals per treatment and sex). Significance was tested by One-Way ANOVA and Bonferroni post hoc test. Data are presented as mean ± SD. Adjusted p values are shown. **d**, Water consumption of female and male mice was not significantly changed. **e**,**f**, Plasma level of Aspartate transaminase (AST) (**e**) and Alkaline Phosphatase (ALP) (**f**) as biomarkers of liver health were not significantly changed. Significance was tested by One-Way ANOVA. N = 5 per treatment for both sexes. Data in d-f are presented as mean ± SEM.

To determine the dose range of trametinib that efficiently inhibited Ras/Mek/Erk signaling without adverse effects on health, young, C3B6F1 hybrid, wild-type male and female mice were fed with 0.29 mg, 0.58 mg, 1.44 mg, 2.88 mg or 11.52 mg of trametinib per kg of diet for 4 weeks (Fig. 1a), and their plasma levels of trametinib (Fig. 1b,c), Ras–Mek–Erk pathway activity (Fig. 1d,e), body weight (Fig. 1f,g) and spleen weight (Fig. 1h,i and Extended Data Fig. 1) were measured. Trametinib in plasma increased with dietary concentrations and was significantly higher in female mice than in male mice (Fig. 1b,c). Inhibition of MEK activity measured by western blot of liver samples using phosphorylation of ERK1/2 (pERK1/2) as read-out (Fig. 1d,e) showed that Erk1/2 phosphorylation was unaffected at 0.29mg kg^−1^ and 0.58 mg kg^−1^ trametinib and reduced at concentrations of 1.44 mg kg^−1^ trametinib and higher in both sexes, although low sample number and high variability meant that only 11.52 mg kg^−1^ in female mice resulted in a significant reduction. Decreased Ras–Mek–Erk pathway activity upon trametinib treatment was also observed in kidney, spleen and muscle tissue (Extended Data Fig. 1a–c). pERK1/2 levels were significantly reduced in kidney and spleen tissue of female mice treated with 1.44 mg kg^−1^ trametinib (Extended Data Fig. 1a,b) with a non-significant trend in female (Extended Data Fig. 1c) and male (Extended Data Fig. 1a–c) muscle. Treatment with 11.52 mg kg^−1^ trametinib strongly reduced pERK1/2 levels in all tested tissues in both sexes (Extended Data Fig. 1a–c). Thus, short-term treatment with trametinib led to a dose-dependent downregulation of Ras–Mek–Erk pathway activity in several tissues. Trametinib did not significantly affect water uptake (Extended Data Fig. 1d,e). Trametinib can cause body weight loss, liver lesions and necrosis and increased alanine aminotransferase (ALT) and alkaline phosphatase (ALP) levels indicative of liver dysfunction. There was no significant effect of any dose of trametinib on plasma levels of AST (Extended Data Fig. 1f,g) or ALP (Extended Data Fig. 1h,i). At doses of 0.29–2.88 mg kg^−1^, trametinib also did not affect body weight (Fig. 1f,g) or spleen size (Fig. 1h,i). In contrast, animals fed with 11.52 mg kg^−1^ trametinib failed to gain body weight in the 4-week measurement period (Fig. 1f,g), with a trend for increased spleen weight in both sexes (Fig. 1h,i). In summary, dietary trametinib concentrations of 1.44 mg kg^−1^ and higher were sufficient to inhibit Ras/Mek/Erk signaling, whereas only the highest dose of 11.52 mg kg^−1^ trametinib induced adverse effects on mouse health. We, therefore, used 1.44 mg kg^−1^ in all subsequent experiments.

### Trametinib and rapamycin additively extend lifespan in mice

**Fig. 2:**
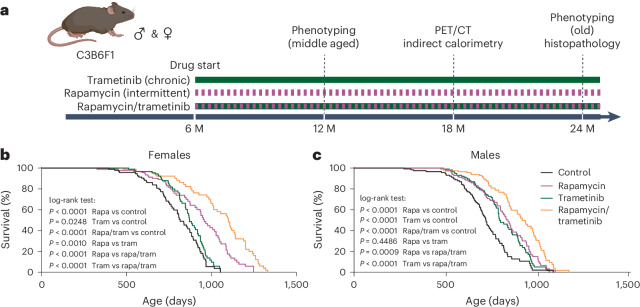
Trametinib alone extends lifespan and acts additively with rapamycin to do so. **a**, Schematic overview of trametinib and rapamycin treatment. **b**,**c**, Survival of female (**b**) and male (**c**) mice. Trametinib significantly increased survival in both sexes. Combined treatment significantly increased lifespan compared to the individual drug treatments (log-rank test). Curves represent pooled survival data from the lifespan, tissue collection and phenotyping cohorts (sample size: control ♂ *n* = 121, ♀ *n* = 100; rapamycin ♂ *n* = 120, ♀ *n* = 98, trametinib ♂ *n* = 121, ♀ *n* = 98; combined ♂ *n* = 120, ♀ *n* = 98). For survival data of the individual cohorts, see Extended Data Fig. 2a–f. Lifespan studies of control and drug-treated animals were performed at the same time within the cohorts. M, months. Source data

Mice were continuously fed with 1.44 mg kg^−1^ trametinib starting from 6 months of age, and their survival was measured (Fig. 2a). Trametinib treatment caused a significant lifespan extension in both sexes: in female mice with a median lifespan extension of 7.2% (*P* = 0.0248, log-rank test) (Fig. 2b) and no significant effect on maximum lifespan (*P* = 0.474, Wang–Allison test) and in male mice with an increase in median lifespan of 10.2% (*P* < 0.0001, log-rank test) (Fig. 2c) and maximum lifespan of 15.8% (*P* = 0.014, Wang–Allison test). Cox proportional hazard analysis showed no significant interaction between sex and trametinib treatment (*P* = 0.1793), showing that trametinib increased survival of male and female mice to a similar extent.

In *Drosophila*, trametinib and rapamycin combine additively to extend lifespan^19^. We measured the survival of mice on diets containing only rapamycin (42 mg kg^–1^), the dose so far inducing the largest increase in longevity^20^, or both rapamycin (42 mg kg^–1^) and trametinib (1.44 mg kg^–1^). Rapamycin was fed in alternate weeks (Fig. 2a), which improves metabolic health parameters^11,21^ and extends lifespan to a similar degree as continuous treatment^22^. As previously shown^22^, intermittent rapamycin treatment extended lifespan in both sexes to a similar extent (*P* < 0.0001, log-rank test; *P* = 0.2481 for the sex–drug interaction, Cox proportional hazard) with an increase in median and maximum lifespan of 17.4% and 16.5%, respectively, in female mice (Fig. 2b) and 16.6% and 18.3%, respectively, in male mice (Fig. 2c). In contrast to the single treatments, combined treatment with rapamycin and trametinib increased survival more in female mice than in male mice (sex–treatment interaction *P* = 0.0218, Cox proportional hazard) and caused a larger increase compared to the single treatment (Fig. 2b,c) in both sexes, with median and maximum lifespan increased by 34.9% and 32.4%, respectively, in female mice (Fig. 2b) and by 27.4% and 26.1%, respectively, in male mice (log-rank test; see Fig. 2b,c for the corresponding *P* values). In female mice, the hazard ratio of trametinib alone compared to control (*P* = 0.0383) was 0.6926 (0.4886–0.9804) and 0.4478 (0.3002–0.6641) in the presence of rapamycin (*P* < 0.0001). Rapamycin alone had a hazard ratio of 0.3284 (0.2243–0.4784) compared to controls (*P* < 0.0001) and 0.2123 (0.1380–0.3234) in the presence of trametinib (*P* < 0.0001). In male mice, trametinib alone had a hazard ratio of 0.5257 (0.3845–0.7169) versus controls (*P* < 0.0001) and 0.5814 (0.4127–0.8154) in the presence of rapamycin (*P* = 0.0017). Rapamycin on its own had a hazard ratio of 0.4623 (0.3376–0.6316) compared to controls (*P* < 0.0001) and 0.5113 (0.3611–0.7208) in the presence of trametinib (*P* = 0.0001). There was, thus, no indication of a positive or negative interaction between drug treatments in either sex.

In summary, treating mice with either trametinib or rapamycin extended lifespan in both sexes, with lifespan further extended by combined treatment in an additive manner.

### Effects of trametinib and rapamycin on mouse health

**Extended Data Fig. 3:**
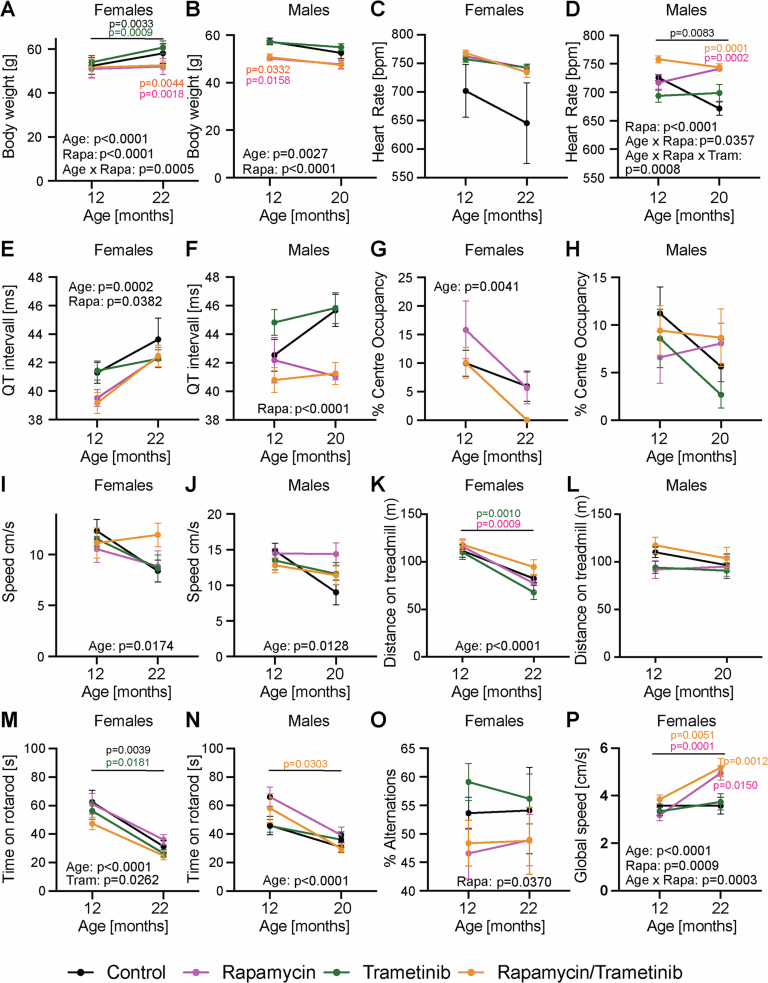
Rapamycin treatment reduces body weight, improves heart function and increases movement speed in old mice. **a**, **b** body weight, **c**–**f** ECG measurement of **c**, **d** heart rate and **e**, **f** QT-interval, **g**–**j** open field assay measurement of **g**, **h** centre occupancy and **i**, **j** movement speed, **k**, **l** distance on treadmill, **m**, **n** time on rotarod, **o**, **p** Y maze measurement of **o** % activity in new arm and **p** global speed. Data were analysed using a mixed effect model testing for the interaction between Rapamycin x Trametinib x Age followed by a Bonferroni post hoc test to test for significance changes between 12 and 22 months indicated by p values above the connecting lines and between drug treatments and control at 12 or 22 months indicated by p values next to the dots. Significant terms from the interaction analysis and the corresponding p-values are indicated per panel. N numbers (12 M, 22 M) for: female body weight, rota rod: Control (15/13), Rapamycin (15/13), Trametinib (15/10) Rapamycin/Trametinib (15/15); male body weight, rota rod: Control (14/10), Rapamycin (15/13), Trametinib (15/13), Rapamycin/Trametinib (15/15); female heart rate, QT-interval: Control (15/9), Rapamycin (15/10), Trametinib (8/10) Rapamycin/Trametinib (7/7); male heart rate, QT-interval: Control (15/9), Rapamycin (15/10), Trametinib (8/10) Rapamycin/Trametinib (7/7). N numbers (22 M female, 20 M male) for: open field: Control (13,11), Rapamycin (14,13), Trametinib (12,13) Rapamycin/Trametinib (15,15); treadmill: Control (13,11), Rapamycin (14,13), Trametinib (12,13), (Rapamycin/Trametinib (15,15).

**Extended Data Fig. 4:**
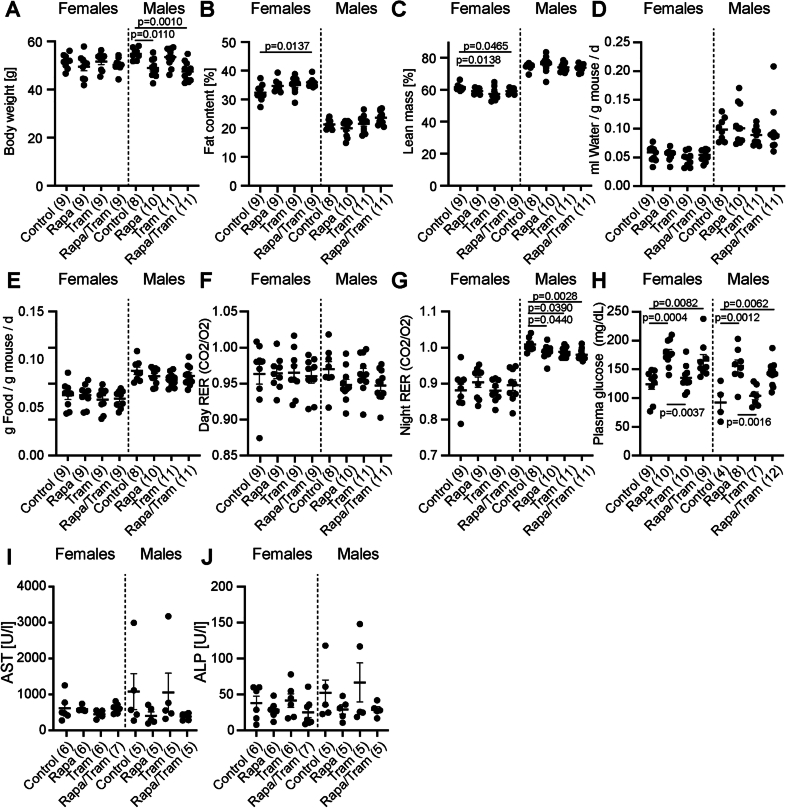
Rapamycin and trametinib do not affect food or water uptake but do affect night time metabolism in males. **a** body weight. **b** nuclear magnetic resonance measurement of **b** % body fat and **c** % lean mass. Metabolic cages were used to measure **d** water uptake **e** food uptake relative to body weight and **f**, **g** respiratory exchange ratio (RER) during **f** day and **g** night. Measurements were done in batches between the age of 15–18 months. Rapamycin and combined treatment significantly reduced body weight in male but not female mice, but had no effect on water or food uptake. Fat content was significantly increased upon combined treatment in females. Lean mass was significantly decreased in trametinib and combined treated females. Males showed no significant differences in fat or lean mass. All drug treatments significantly reduced night-time RER in males but not females. **h** glucose, **i** aspartate transaminase (AST) and **j** alkaline phosphatase (ALP) level in blood plasma of 24 months old animals. Rapamycin but not trametinib significantly increased plasma glucose levels in male and female mice. Combination of trametinib and rapamycin did not further increase plasma glucose levels compared to rapamycin treated animals. One-Way ANOVA with Bonferroni post hoc test. Numbers in brackets indicate the number of plasma samples of different animals assessed per treatment. Data are presented as mean ± SEM.

We measured health parameters, including body weight (Extended Data Fig. 3a,b), heart function (Extended Data Fig. 3c–f), anxiety behavior (Extended Data Fig. 3g–j), endurance (Extended Data Fig. 3k,l), motor coordination (Extended Data Fig. 3m,n) and memory (Extended Data Fig. 3o,p), at middle age (12 months) and old age (20–22 months). In addition, we used metabolic cages to measure body weight (Extended Data Fig. 4a), fat and lean mass (Extended Data Fig. 4b,c), water and food uptake (Extended Data Fig. 4d,e) and respiratory exchange ratio (RER) (Extended Data Fig. 4f,g) at 16 months of age. Consistent with the pre-test, 1.44 mg kg^−1^ trametinib had no effect on body weight (Extended Data Figs. 3a,b and 4a). In contrast, both rapamycin and combined drug treatment decreased body weight in female mice at 20 months (Extended Data Figs. 3a and 4a) and in male mice at 12, 16 and 20 months (Extended Data Figs. 3b and 4a). Combined treatment induced a significant increase in fat content (Extended Data Fig. 4b) and a decrease in lean mass (Extended Data Fig. 4c) in female mice but not male mice. Lean mass was lower in female mice treated with trametinib (Extended Data Fig. 3c). The drug treatments did not affect water or food uptake (Extended Data Fig. 4d,e). Heart rate of both sexes declined with age, and, in male mice, both rapamycin and trametinib attenuated the decline, with a similar non-significant trend in female mice (Extended Data Fig. 3c,d). QT interval, a read-out for left ventricular hypertrophy, increased with age, and rapamycin decreased QT interval in female mice, and both rapamycin and combined treatment prevented the age-related increase in male mice (Extended Data Fig. 3e,f). The drug treatments did not affect center occupancy in the open field (Extended Data Fig. 3g,h), forced running endurance on a treadmill (Extended Data Fig. 3k,l) or motor coordination in a rotarod assay (Extended Data Fig. 3m,n). Rapamycin-treated female mice performed slightly worse in the Y-maze (Extended Data Fig. 3o). Rapamycin-treated and combination-treated male mice showed increased global speed in the Y-maze (Extended Data Fig. 3p) with a similar trend for the combined drug treatment in the open field (Extended Data Fig. 3i), suggesting a higher motivation to move at old age. RER was not significantly altered by drug treatment in 16-month-old female mice (Extended Data Fig. 4f,g), whereas all three drug treatments significantly reduced RER during the night (Extended Data Fig. 4g), indicating a slight shift toward mixed carbohydrate and lipid utilization.

Chronic rapamycin treatment negatively affects glucose homeostasis and can lead to hyperglycemia. To assess the effect of trametinib, we measured glucose levels in plasma of 24-month-old animals. Both male and female mice treated with rapamycin showed significantly increased plasma glucose levels (Extended Data Fig. 4h). In contrast, plasma glucose levels of trametinib-treated animals did not differ significantly from controls (Extended Data Fig. 4h). Combination-treated mice had plasma glucose levels similar to rapamycin-treated animals, indicating that trametinib did not further exacerbate the effect of rapamycin. Thus, in contrast to rapamycin, trametinib extends lifespan without causing hyperglycemia. In human patients, trametinib treatment can result in increased plasma levels of ALT and ALP, indicative of reduced liver function. In contrast, treating mice chronically with 1.44 mg of trametinib did not change plasma levels of AST and ALT in 24-month-old animals (Extended Data Fig. 4i), consistent with the results from young animals (Extended Data Fig. 1f–i). Prolonged treatment with this dose of trametinib, therefore, did not affect liver function in mice.

In summary, rapamycin treatment alone or in combination with trametinib reduced body weight in both sexes and attenuated the decline in heart function with age. Both rapamycin and trametinib attenuated the age-related decline in heart rate in male mice; rapamycin decreased QT interval in both sexes; and combined treatment prevented the age-related increase in male mice. Rapamycin-treated and combination-treated female mice moved more at late age, and all drug treatments reduced nighttime RER in male mice. Rapamycin, but not trametinib, treatment caused hyperglycemia in old animals.

### Combined drug treatment delays tumor growth

**Fig. 3:**
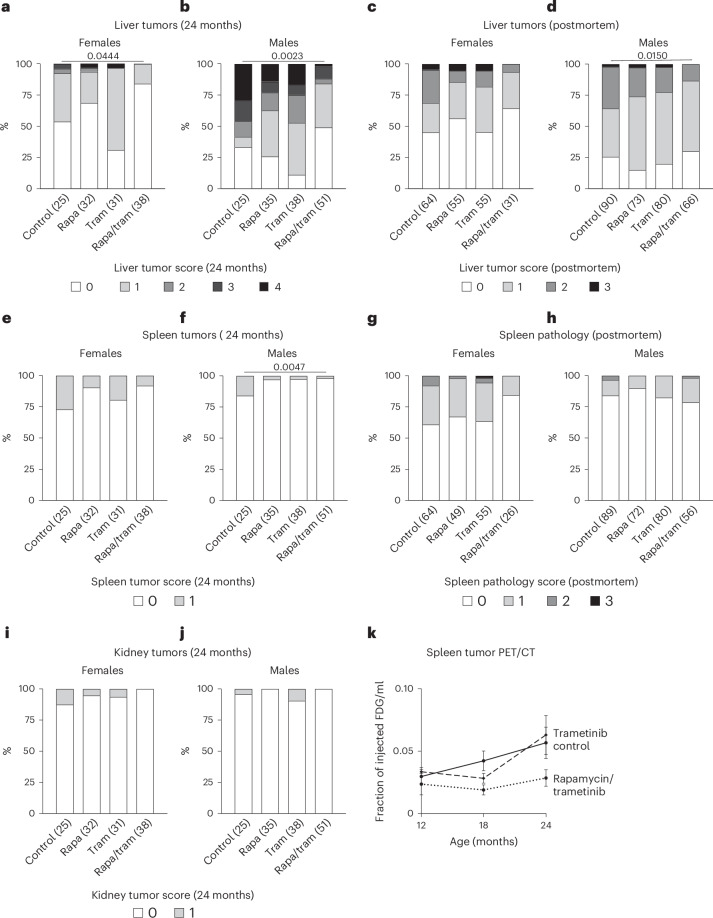
Trametinib and rapamycin combined treatment delays tumor formation and overall tumor load. Cross-sectional histopathological analysis of tumor levels in liver (**a**,**b**), spleen (**e**,**f**) and kidney (**i**,**j**) samples of 24-month-old female mice (**a**,**e**,**i**) and male mice (**b**,**f**,**j**). Combined drug treatment significantly reduced liver tumors in both sexes at 24 months and spleen tumors in male mice, with a similar trend for reduced tumor load in female spleen and male and female kidney (**e**,**f**). The absence of liver tumors was scored with 0, and the presence of low, moderate, high and very high severity tumors was scored with 1, 2, 3 and 4, respectively. The absence or presence of spleen and kidney tumors was scored with 0 or 1, respectively. Liver tumor data for rapamycin and control animals were previously published^22^. Postmortem macropathology analysis of liver (**c**,**d**) and spleen (**g**,**h**) of animals from the lifespan and phenotyping cohort. **k**, Longitudinal analysis of spleen tumor progression via ^18^F-FDG PET/CT measurements in the same female animals at 12, 18 and 24 months of age. Although tumor load increased from 12 months to 24 months in control and trametinib-treated mice, combination-treated animals showed no increase. Data are presented as mean ± s.e.m. ^18^F-FDG uptake of rapamycin/trametinib versus control at 24 months *P* = 0.08. Two-way ANOVA with post hoc Bonferroni test and simple linear regression. (Control *n* = 7, trametinib *n* = 5 and rapamycin/trametinib *n* = 4). Data in **a**–**j** are presented as percentage over total, and statistical analysis was performed by two-sided chi-square test and Poisson regression. Numbers in brackets indicate the number of scored tissues per treatment.

Trametinib and rapamycin are cancer chemotherapeutics. To address whether their lifespan-extending effects were associated with their anti-cancer function, we performed a cross-sectional histopathological analysis at 24 months of age, complemented by a macropathological assessment of overall tumor load postmortem, to assess any delay in tumor formation. Untreated mice predominantly developed tumors in the liver (Fig. 3a–d), spleen (Fig. 3e–h) and kidney (Fig. 3i,j). Liver tumors were most prevalent, with approximately 50% of female mice (Fig. 3a) and 70% of male mice (Fig. 3b) with tumors at 24 months of age. No significant differences in liver tumors were detected with either trametinib or rapamycin treatment at 24 months (Fig. 3a,b) or postmortem (Fig. 3c,d). However, combination treatment significantly reduced liver tumor numbers in both sexes at 24 months of age (Fig. 3a,b) and in male mice postmortem (Fig. 3d), with a similar trend in female mice (Fig. 3c). Twenty-five percent of female mice and 20% of male mice had tumors in the spleen (Fig. 3e,f), and 15% of female mice and 5% of male mice showed kidney tumors (Fig. 3i,j) at 24 months of age. Neither trametinib nor rapamycin reduced tumor formation in the spleen (Fig. 3e,f) or the kidney (Fig. 3i,j). The combination treatment significantly reduced spleen tumor formation at 24 months of age in male mice (Fig. 3f), with a similar trend in female mice (Fig. 3e). Although we did not detect any tumors in the kidney upon combined treatment, this tumor type was so rare in controls that the effect was not significant. In summary, only the combination of rapamycin and trametinib caused significant effects on tumor load, and this was more obvious in the cross-sectional analysis—for example, compare spleen pathology in male mice at 24 months and postmortem (Fig. 3f versus Fig. 3g), which may indicate a delay in tumor formation rather than reduced overall lifetime tumor load. To further investigate, we performed a longitudinal analysis, measuring tumor formation in vivo by ^18^F fluodeoxyglucose (^18^F-FDG) positron emission tomography (PET) coupled with computed tomography (CT)^23^ in the same animals at 12, 18 and 24 months of age (Fig. 3k). Because the capacity for the PET/CT measurement was limited, we restricted our analysis to control, trametinib-treated and combination-treated female mice. Tumors were identified as hypermetabolic regions, first in 24-month-old animals and then traced back in the same animals at 18 months and 12 months of age. Tumors were detected in several organs, including liver and uterus, but could only be reliably quantified in the spleen (Fig. 3g). Tumor occurrence increased with age in control animals, and, consistent with the histopathological analysis (Fig. 3e), trametinib did not affect spleen tumors at 12, 18 or 24 months (Fig. 3k). In contrast, combination-treated animals showed lower spleen tumors at all three ages, with no significant increase with age. Combined drug treatment, thus, delayed tumor formation, but, due to the missing single rapamycin treatment, the effect may have been entirely attributable to rapamycin.

### Combined drug treatment attenuates age-related brain glucose uptake

**Fig. 4:**
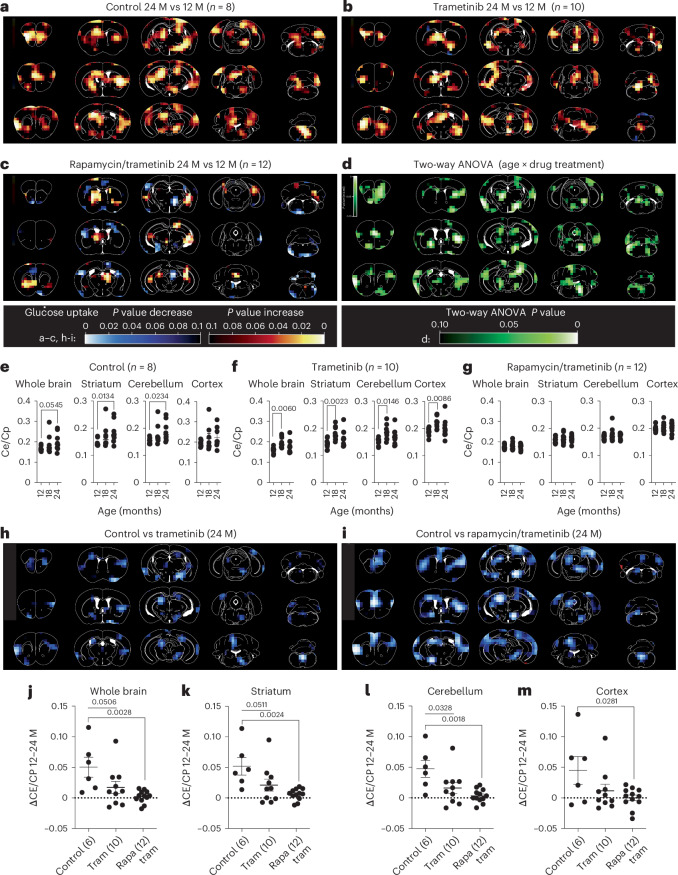
Combined treatment with trametinib and rapamycin counteracts the age-related increase in brain glucose uptake. Longitudinal ^18^F-FDG PET/CT measurements of age-related changes in differential glucose uptake in the female mouse brain. **a**–**c**, Images show differential regional glucose uptake across the mouse brain between 24-month-old and 12-month-old control (**a**), trametinib-treated (**b**) and rapamcin/trametinib-treated (**c**) female mice. Color code represents the *P* value for the indicated voxels in a paired Student’s *t*-test comparing 24-month-old to 12-month-old animals. Increase in glucose uptake is shown in red/yellow, decrease in blue color. **d**, Two-way ANOVA analysis highlights brain regions that showed a significant interaction between age and drug treatment in differential glucose uptake. A lighter green color indicates a higher significance in the two-way ANOVA analysis. **e**,**f**, Longitudional quantification of glucose uptake in whole brain, striatum, cerebellum and cortex at 12, 18 and 24 months of age in control (**e**), trametinib-treated (**f**) and rapamycin/trametinib-treated (**g**) mice. Glucose uptake significantly increased with age in striatum and cerebellum of control animals but not in combination-treated animals. Data are presented as mean ± s.e.m. **h**,**i**, Images show differential glucose uptake in the brain of 24-month-old trametinib versus control (**h**) and combination versus control (**i**) mice. **j**–**m**, Quantification of differential glucose uptake between 12-month-old and 24-month-old whole brain (**j**), striatum (**k**), cerebellum (**l**) and cortex (**m**). Decrease in glucose uptake is indicated in blue color. Age-related differences in glucose uptake between 12 months and 24 months were significantly lower in combination-treated animals compared to controls. A similar but milder effect was also observed in trametinib-treated animals, which was only significant in the cerebellum. Two-way ANOVA with Bonferroni post hoc test. Data are presented as mean with s.e.m. **a**–**g** and **j**–**m**, Numbers in brackets indicate the number of whole brains/brain regions analyzed per treatment and timepoint. M, months.

Brain glucose uptake increases during mouse aging^24^ and may be linked to impaired cognitive function^25^. We, therefore, used the ^18^F-FDG PET/CT longitudinal data to address whether trametinib and combined drug treatment attenuated age-related increase in glucose uptake (Fig. 4a–g). ^18^F-FDG uptake increased with age in control female mice across the whole brain (Fig. 4a,e), consistent with a previous study^24^. ^18^F-FDG uptake increased significantly between 12 months and 24 months in the striatum and cerebellum but not the cortex (Fig. 4a,e). Uptake in trametinib-treated female mice showed a significant increase across the whole brain and in the striatum, cerebellum and cortex between 12 months and 18 months of age (Fig. 4b,f), with no further increase between 18 months and 24 months. Combined drug treatment prevented the age-related increase in glucose uptake both across the whole brain and in the striatum, cortex and cerebellum (Fig. 4c,g), confirmed by two-way ANOVA analysis (*P* < 0.05 interaction between age and drug treatment; Fig. 4d). Direct comparisons of uptake between 12 months and 24 months (Fig. 4f–h) showed that trametinib treatment induced an intermediate phenotype between control and combination-treated animals with an increase in glucose uptake, whereas there was no change in the double drug-treated mice. Trametinib treatment, thus, ameliorated the age-related increase in brain glucose uptake but only completely blocked it when combined with rapamycin.

### Combined drug treatment reduces brain inflammation in old mice

**Fig. 5:**
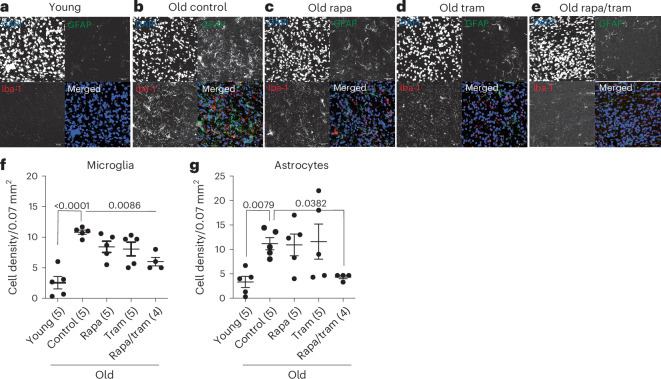
Combination treatment attenuates age-related microglia activation and astrocyte density in the striatum. **a**–**e**, Representative confocal composite images of immunohistochemical stainings of the striatum region in 6-month-old young controls (**a**) and 24-month-old controls (**b**) and 24-month-old rapamycin-treated (**c**), 24-month-old trametinib-treated (**d**) and 24-month-old rapamycin/trametinib-treated (**e**) female mice. Nuclei were stained with DAPI, astrocytes with GFAP and activated microglia with Iba-1. **f**,**g**, Quantification of microglia density (DAPI/Iba-1^+^ cells) (**f**) and astrocyte density (DAPI/GFAP^+^ cells) (**g**) per mouse striatum of young and old control animals and old drug-treated animals. Microglia and astrocyte density significantly increased with age in the striatum of control animals. Combination treatment significantly reduced microglia and astrocyte density in the striatum compared to old controls. One-way ANOVA with Bonferroni post hoc test. 10–15 confocal images per brain region were averaged per sample. Numbers in brackets indicate the number of brain regions of individual animals analyzed per treatment. Data are presented as mean ± s.e.m.

**Extended Data Fig. 5:**
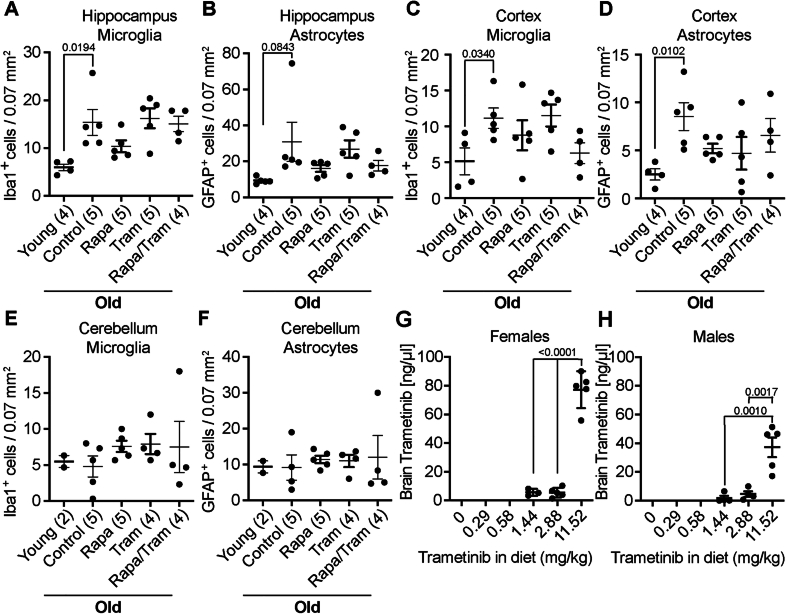
Combined rapamcin and rametinib treatment affects brain inflammation in a region-specific manner. Quantification of immunohistochemical stainings measuring the density of activated microglia (DAPI/Iba-1 positive cells) and astrocyte (DAPI/GFAP positive cells) in **a**, **b** hippocampus, **c**, **d** cortex and **e**, **f** cerebellum of 6-months old control animals (young) and 24 months-old control and drug-treated females. Microglia and astrocyte density significantly increased with age in the hippocampus and cortex of control animals, but not in the cerebellum. Single drug treatment did not significantly reduce microglia or astrocyte density in the hippocampus, cortex or cerebellum. **g**, **h** Brain levels of trametinib increased in a dose-dependent manner upon feeding **g** female or **h** male animals for 4 weeks with food containing 0, 0.29, 0.58, 1.44, 2.88 or 11.52 mg/kg of drug. N = 5 brain samples were measured via mass spectrometry per treatment. Only samples with significant trametinib levels above background are shown. Trametinib was undetectable in the brain of animals fed with 0, 0.29 and 0.58 mg/kg trametinib, and only very low levels were detected in animals fed with 1.44 and 2.88 mg/kg trametinib. Only at 11.52 mg/kg trametinib, higher levels of trametinib were detected in the brain. One-Way ANOVA with Bonferroni post hoc test. 10–15 confocal images per brain region were averaged per sample in **a**–**f**. Numbers in brackets indicate the number of brain regions of different animals analyzed per treatment. Data are presented as mean ± SEM.

Brain inflammation increases the requirements for glucose and results in greater ^18^F-FDG uptake^26^. Microglia are the primary immune cells of the brain, and their increased activity is associated with age-related brain inflammation accompanied by synapse loss and late-onset neurodegeneration^27^. Microglia act in concert with astrocytes to resolve inflammation, and astrocyte-dependent Tgfβ secretion can inhibit microglia activation^28^. Aging disrupts this interplay, leading to exacerbated inflammation in both an autonomous and an astrocyte-dependent manner^29^. Because Ras/MEK/Erk signaling has been implicated in microglial activation^27^, we assessed whether drug treatment reduced activation of microglia and astrocyte density by performing immunohistological stainings of brain sections using antibodies against Iba-1 and Gfap for activated microglia and astrocyte density, respectively (Fig. 5a–e), in brains of 24-month-old female control (Fig. 5b), rapamycin-treated (Fig. 5c), trametinib-treated (Fig. 5d) and rapamycin/trametinib-treated (Fig. 5e) mice. We also stained brains of 6-month-old control mice (Fig. 5a). Activated microglial and astrocyte density significantly increased in the striatum (Fig. 5f,g), hippocampus (Extended Data Fig. 5a,b) and cortex (Extended Data Fig. 5c,d) but not the cerebellum (Extended Data Fig. 5e,f) of 24-month-old compared to 6-month-old controls, indicating increased brain inflammation at older age. Combined drug treatment significantly reduced the density of activated microglia and astrocytes in the striatum of 24-month-old mice (Fig. 5f,g), with a similar trend in the cortex (Extended Data Fig. 5c,d) but no significant effects in the hippocampus (Extended Data Fig. 5a,b) or the cerebellum (Extended Data Fig. 5e,f). Compared to the combined treatment, single treatment with rapamycin or trametinib did not significantly reduce the number of activated microglia in the striatum (Fig. 5f). Thus, rapamycin and trametinib had only mild effects, but the combination of both drugs significantly reduced age-related brain inflammation in specific regions. Notably, ^18^F-FDG uptake was increased in the cerebellum, suggesting mechanisms other than inflammation.

As Ras/MEK/Erk signaling has been implicated in microglia activation^27^, we assessed whether trametinib crossed the blood–brain barrier. Trametinib levels were measured in the brains of the animals used for the pre-test (Extended Data Fig. 5g,h). At dietary concentrations of 0.29 mg kg^−1^ and 0.58 mg kg^−1^, trametinib was not detected in the brain (Extended Data Fig. 5g,h). Significant, but very low, levels of trametinib were detected at 1.44 mg kg^−1^ and 2.88 mg kg^−1^ dietary concentrations, with levels 25-fold and 30-fold lower than the corresponding plasma concentrations of female (140 ng µl^−1^) and male (55.8 ng µl^−1^) mice, respectively. Trametinib at 1.44 mg kg^−1^ thus barely crossed the blood–brain barrier. Only at 11.52 mg kg^−1^ did we detect trametinib in the brain, which might indicate saturation of the efflux system due to the high trametinib plasma levels. In conclusion, the effects on microglia upon treatment with 1.44 mg kg^−1^ trametinib might be indirect and caused by the action of trametinib in peripheral tissues.

### Combined drug treatment reduces peripheral inflammation

**Fig. 6:**
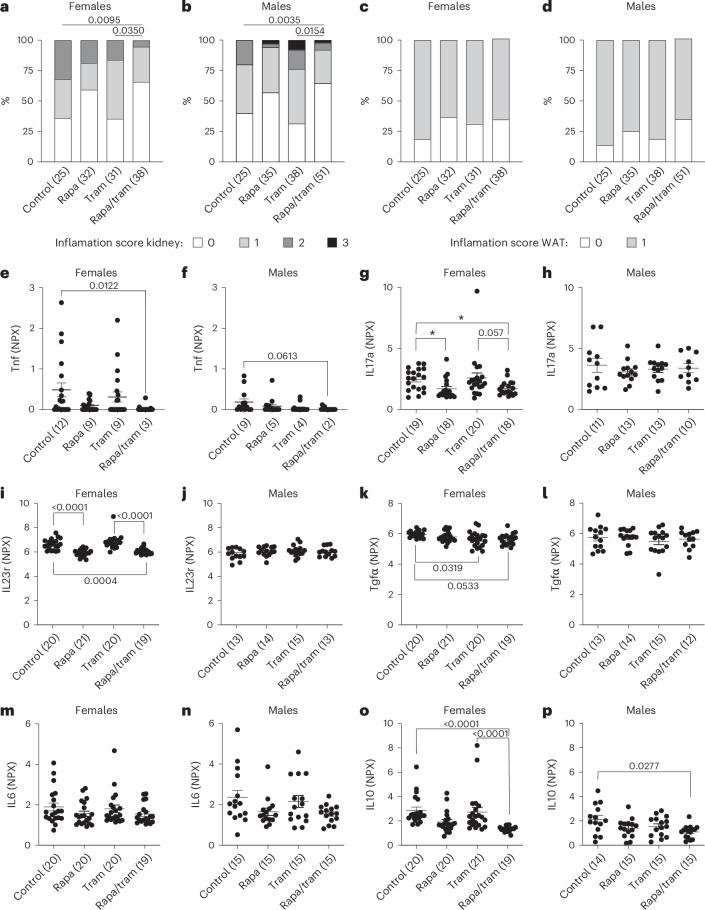
Combination drug treatment reduces peripheral inflammation. **a**–**d**, Histopathological analysis of inflammation in kidney (**a**,**b**) and WAT (**c**,**d**) of 24-month-old female mice (**a**,**c**) and male mice (**b**,**d**). Combination-treated female mice and male mice showed significantly reduced kidney inflammation compared to control and trametinib-treated animals. Linear regression. Numbers in brackets indicate the number of tissue samples from independent mice per treatment. There was a non-significant trend for reduced WAT inflammation upon treatment with rapamycin and rapamycin/trametinib. **e**–**p**, Plasma protein levels of circulating cytokines as measured by the Olink Target 96 Mouse Exploratory panel: Tnf (**e**,**f**), IL17a (**g**,**h**), IL23r (**i**,**j**), Tgfα (**k**,**l**), IL6 (**m**,**n**) and IL10 (**o**,**p**). Drug effects on plasma cytokine levels were highly sex specific, with Tnf, IL17a, IL23r and Tgfα being downregulated by the combination treatment only in female mice but not male mice. One-way ANOVA with Tukeyʼs post hoc test. Numbers in brackets indicate the number of plasma samples from independent mice per treatment. Data in **e** and **p** are presented as mean ± s.e.m. Inflammation and plasma cytokine data for control and rapamycin-treated animals were previously published^22^.

During aging, inflammation increases in multiple peripheral organs, manifested in a low-level, systemic, pro-inflammatory phenotype termed ‘inflammaging’^30^. Inflammaging is associated with both mTOR and Erk pathway activity^31^. Combined treatment with rapamycin and trametinib reduced brain inflammation, so we assessed inflammation in peripheral tissues by histopathology of kidney and white adipose tissue (WAT) (Fig. 6a–d). Controls showed high levels of kidney inflammation, with 64% of female mice and 60% of male mice affected at 24 months of age (Fig. 6a,b). Trametinib did not reduce kidney inflammation (Fig. 6a,b), with a non-significant reduction with rapamycin treatment (40% of female mice and 43% of male mice affected at 24 months). Combined treatment significantly reduced kidney inflammation in both sexes with only 34% of female mice and 35% of male mice affected. Most of the combined drug effect was, therefore, probably due to rapamycin, which positively affects kidney health^32^. Control mice also showed high levels of inflammation in the WAT, with 82% of female mice and 86% of male mice affected (Fig. 6c,d). No drug treatment significantly reduced WAT inflammation, with 69%, 63% and 65% of female mice and 81%, 76% and 65% of male mice affected in trametinib-treated, rapamycin-treated and combination-treated animals, respectively.

We also assessed inflammatory markers in plasma of both sexes at 24 months, using the Olink Target 96 Mouse Exploratory panel (Fig. 6e–l and Supplementary Table 1). Combination-treated female mice at 24 months showed significantly reduced plasma levels of key pro-inflammatory proteins, including tumor necrosis factor (Tnf; Fig. 6e), interleukin-17a (IL17a; Fig. 6g), interleukin-23r (IL23r; Fig. 6i) and transforming growth factor α (Tgfα; Fig. 6k). Trametinib-treated female mice showed a significant reduction only in Tgfα (Fig. 6k), and rapamycin treatment significantly reduced levels of IL17a (Fig. 6g) and IL23r (Fig. 6i). The single-drug treatments had no significant effect on pro-inflammatory cytokines in male mice, with only interleukin-6 (IL6), which was not regulated in female mice (Fig. 6m), showing slight downregulation (Fig. 6n). Tnf (Fig. 6f) and IL6 levels (Fig. 6n) were mildly reduced in combination-treated male mice. Thus, combination treatment reduced the measured pro-inflammatory cytokines only in female mice. Notably, combination treatment significantly reduced the level of the anti-inflammatory cytokine interleukin-10 (IL10) in both female mice (Fig. 6o) and male mice (Fig. 6p), possibly a response to reduced inflammation in these animals. In summary, combination treatment reduced inflammation in peripheral tissues accompanied by reduced levels of circulating pro-inflammatory cytokines.

### Trametinib does not cause side effects induced by rapamycin

**Extended Data Fig. 6:**
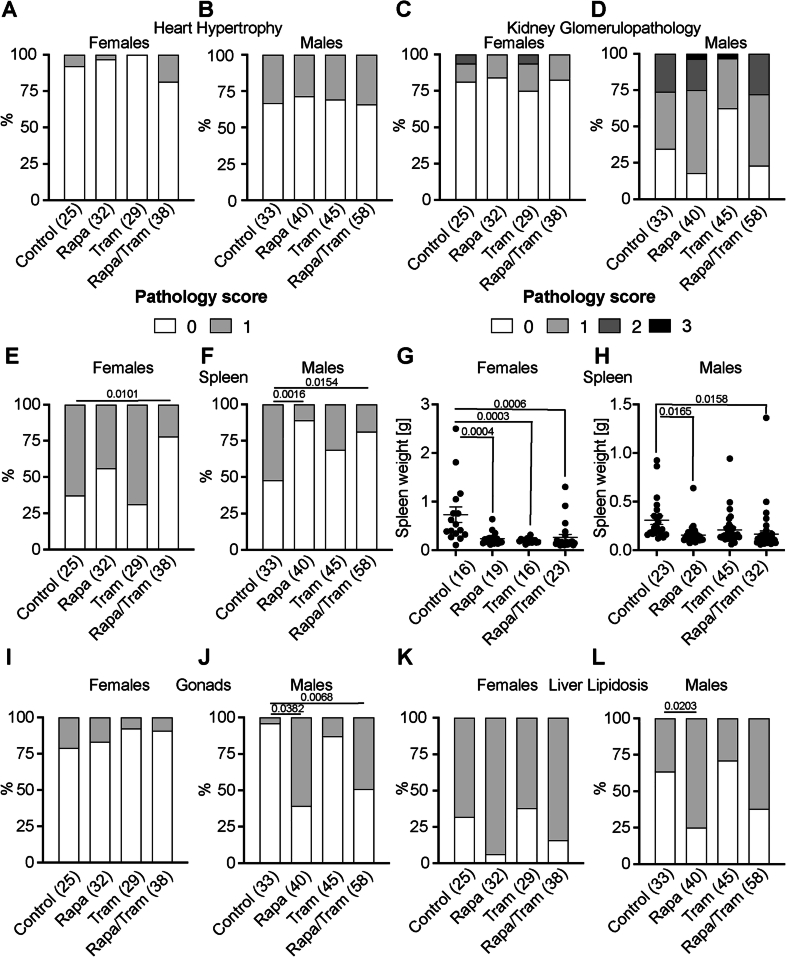
Combination of rapamycin and trametinib ameliorates spleen pathology but does not protect against rapamycin-induced liver lipidosis and testicular lesions. Cross sectional histopathological analysis of non-neoplastic lesions in tissues isolated from 24 months old male and female mice treated with rapamycin, trametinib and rapamycin/trametinib. **a**, **b** heart hypertrophy, **c**, **d** spleen pathology, **e**, **f** spleen weight, **g**, **h** kidney glomerulopathology, **i**, **j** gonadal pathology and **k**, **l** liver lipidosis. **a**, **b** Rapamycin and Trametinib treatments did not significantly affect heart hypertrophy. **c**, **d** Females and males treated with Rapamycin/Trametinib showed reduced spleen pathology and a similar trend was observed upon Rapamycin treatment, which was significant in males. **e**, **f** Spleen weight was significantly reduced upon Rapamycin and Rapamycin/Tram treatment in males and females and upon trametinib treatment in females. **g**, **h** Kidney glomerulopathology was not significantly affected by the drug treatments. **i**, **j** Males but not females treated with Rapamycin and Rapamycin/Trametinib showed a significant increase in gonadal pathology. **k**, **l** Male mice treated with Rapamycin showed a significant increase in liver lipidosis compared to control animals. A similar non-significant trend was also observed in female animals and upon Rapamycin/Trametinib treatment in males and females. Data in **a**–**d** and **g**–**l** are presented as percentage over total and statistical analysis was performed by Chi-square test and Poisson regression. Data in **g**, **h** are plotted as mean with SEM and statistical analysis was done using One-Way ANOVA followed by Bonferroni post hoc test. Number of scored tissues per treatment are indicated in brackets. Control and Rapamycin data in **a**–**l** were published previously^22^.

We assessed the impact of the drug treatments on age-related, non-neoplastic pathologies by measuring heart hypertrophy and kidney and spleen pathology at 24 months of age (Extended Data Fig. 6). Heart hypertrophy and kidney glomerulopatholgy were more pronounced in male mice than female mice, but there was no significant effect of any of the drug treatments (Extended Data Fig. 6a–d). Trametinib had no significant effect on spleen pathology in female mice, with a mild reduction in male mice (Extended Data Fig. 6e,f). Rapamycin mildly reduced spleen pathology in female mice, with a significant reduction in male mice (Extended Data Fig. 6e,f). Combined drug treatment significantly reduced spleen pathology in both sexes (Extended Data Fig. 6e,f). Increased spleen size in old mice reflects disrupted microarchitecture, extramedullary hematopoiesis, chronic inflammation and accumulation of senescent cells^33^, all of which impair spleen function. Spleen weight was significantly decreased by trametinib in female mice, with a similar trend in male mice, and by rapamycin and combination treatment in both sexes (Extended Data Fig. 6g,h), suggesting that both drugs combat spleen immunosenescence.

Long-term administration of rapamycin is associated with negative side effects, including liver lipidosis and testicular degeneration^22^. In contrast to rapamycin, trametinib treatment did not induce liver lipidosis or gonadal lesions (Extended Data Fig. 6i–l), whereas animals treated with both drugs showed similar levels to rapamycin-treated mice (Extended Data Fig. 6i–l). Thus, trametinib treatment did not induce these phenotypes, but nor did it protect rapamycin-treated mice from them.

### Transcriptional response to drug treatment is tissue, drug and sex specific

**Fig. 7:**
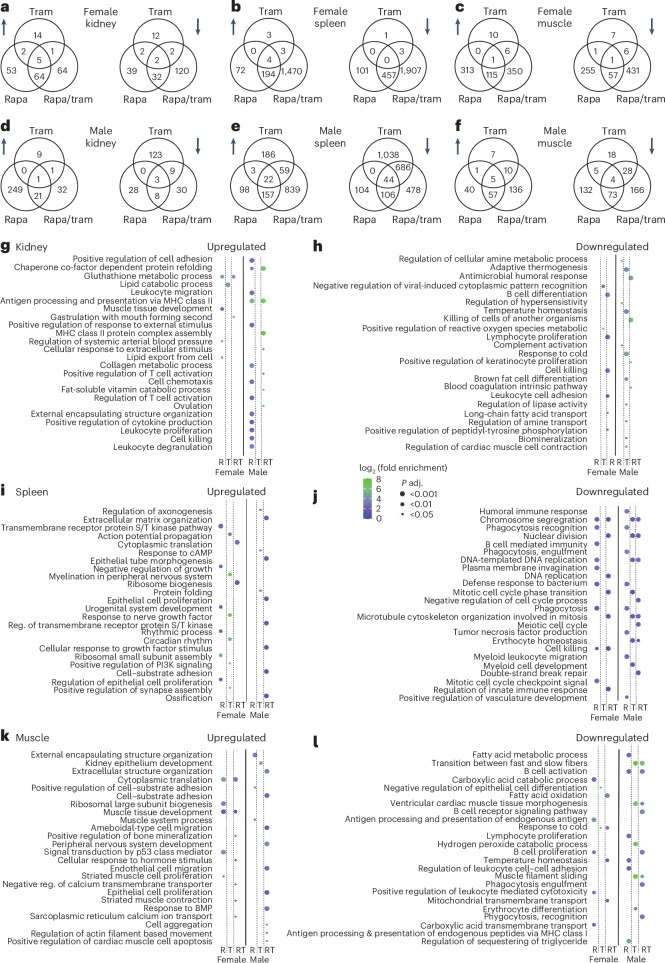
The transcriptome responds in a highly tissue-specific, drug-specific and sex-specific manner toward rapamycin and trametinib treatment. RNA-seq analysis of kidney, spleen and muscle tissues isolated from 24-month-old male and female mice used to measure gene expression changes in response to rapamycin, trametinib and rapamycin/trametinib treatment. **a**–**f**, Venn diagrams depicting the overlap in differential gene expression upon rapamycin, trametinib and rapamycin/trametinib treatment in kidney (**a**,**d**), spleen (**b**,**e**) and muscle (**c**,**f**) tissue. The left Venn diagram per panel shows the overlap in upregulated genes (upward arrow), and the right panel shows downregulated genes (downward arrow). **g**–**l**, GO enrichment analysis was performed using the DAVID API-based function DAVIDenrich of the AGEpy Python package on significantly differential expressed genes upon rapamycin, trametinib and rapamycin/trametinib treatment. Dot plot representation of significantly enriched top GO terms sorted by adjusted *P* value (Benjamini–Hochberg) for upregulated (**g**,**l**,**k**) and downregulated (**h**,**j**,**l**) genes in kidney (**g**,**h**), spleen (**i**,**j**) and muscle (**k**,**l**). The color of the dots indicates log_2_ fold enrichment; the size of the dots indicates the adjusted *P* value for enrichment of the specific GO term. *n* = 4–5 per drug treatment. MHC, major histocompatibility complex; R, rapamycin; T, trametinib.

**Extended Data Fig. 7:**
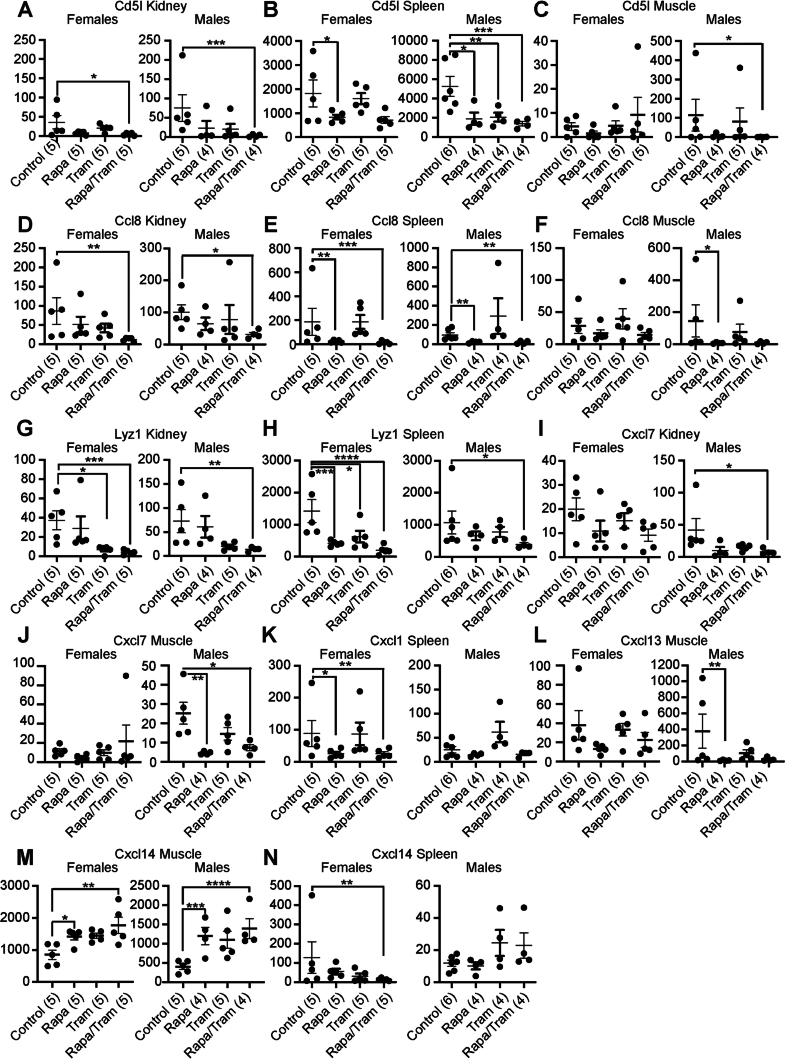
Genes involved in inflammation are downregulated upon combined rapamycin and trametinib treatment. Expression level of inflammation associated genes based on the RNA seq analysis of kidney, spleen and muscle tissue from 24-months-old rapamycin, trametinib and combination treated animals. **a**–**c** *Cd5l* was significantly downregulated in **a** kidney, **b** spleen and **c** muscle upon combination treatment. **d**, **e** *Ccl8* was significantly downregulated by combination treatment in **d** kidney and by rapamcin and combination treatment in **e** spleen and **f** male muscle. **g**, **h** Lyzosyme1 (*Lyz1*) expression was decreased in **g** kidney upon trametinib and combination treatment, and **h** in female spleen upon rapamycin, trametinib and combination treatment. **i**, **j** *Cxcl7* was significantly downregulated in **i** male kidney and **j** male muscle upon combination treatment, and by rapamycin in male muscle. **k** *Cxcl1* expression level was decreased in the female spleen upon rapamycin and combination treatment. **l** *Cxcl13* was significantly downregulated by rapamycin in male muscle tissue. **m**, **n** Expression of *Cxcl14* was upregulated by rapamycin and the combination treatment in **m** male and female muscle. **n** *Cxcl14* was significantly downregulated in the female spleen. Data are presented as Mean ± SEM. Statistical analyses were performed using DESeq2/1.24.0. P-values were adjusted for multiple testing. *P < 0.05 and **P < 0.01, ***P < 0.001, and ****P < 0.0001. Number of scored tissues per treatment are indicated in brackets.

**Extended Data Fig. 8:**
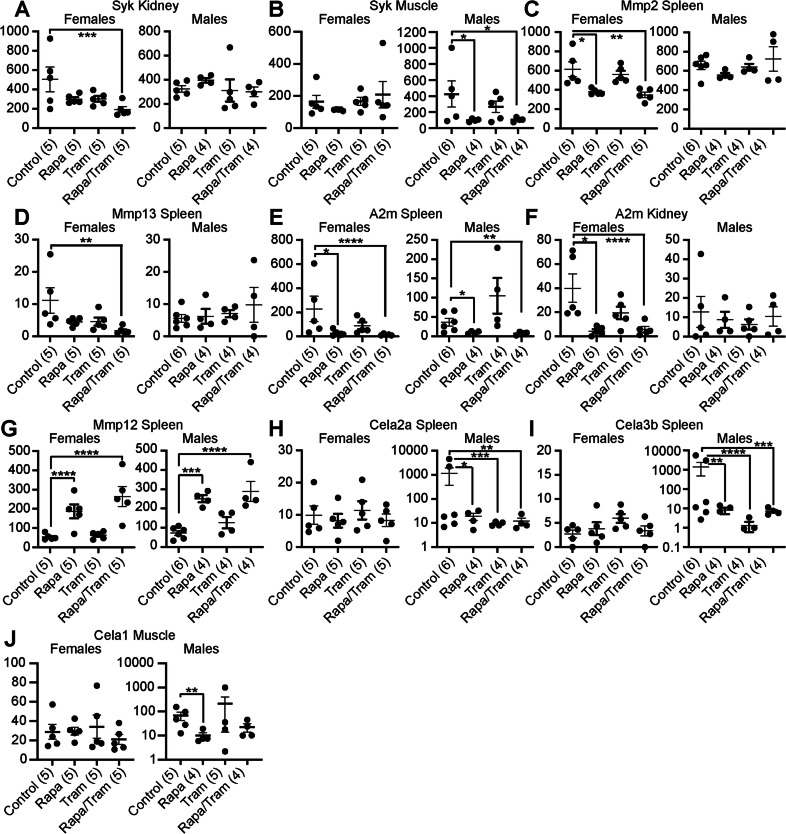
Ets2 target genes are downregulated upon combined rapamycin and trametinib treatment. Expression level of potential ETS target genes based on the RNA seq analysis of kidney, spleen and muscle tissue from of 24-months-old rapamycin, trametinib and combination treated animals. **a** Expression of *SYK* was significantly decreased in the kidney of female mice treated with rapamycin and trametinib, and **b** in male muscle upon rapamycin and combination treatment. **c** *Mmp2* was downregulated in the female spleen upon rapamycin and combination treatment, **d** *Mmp13* expression was decreased in the female spleen upon combination treatment. **e**, **f** *A2m* expression levels were decreased upon rapamycin and combination treatment in **e** male and female spleen and **f** female kidney. **g** *Mmp12* levels were increased upon rapamycin and combination treatment in the spleen. **h** *Cela2a* and **i** *Cela3b* levels were specifically downregulated in male spleens upon single and combination drug treatment. **j** *Cela1* expression was downregulated in male muscle upon rapamycin treatment. Data are presented as Mean ± SEM. Statistical analyses were performed using DESeq2/1.24.0. P-values were adjusted for multiple testing. *P < 0.05 and **P < 0.01, ***P < 0.001, and ****P < 0.0001. Number of scored tissues per treatment are indicated in brackets.

Combination drug treatment reduced age-related kidney inflammation and spleen size. Neutrophils infiltrate muscle in aging mice^34^. We, therefore, measured gene expression changes in response to drug treatment in kidney, spleen and muscle of 24-month-old mice using RNA sequencing (RNA-seq) (Fig. 7 and Supplementary Table 2). In all three organs, the most strongly regulated genes were linked to inflammatory processes (Extended Data Figs. 7 and 8), with the number of differentially expressed genes (DEGs) strongly dependent on drug treatment and tissue type (Fig. 7a–f). Trametinib had only minor effects in female and male kidney (40/146 DEGs) and muscle (32/78 DEGs) and in the female spleen (14 DEGs), whereas, in male spleen, 2,038 genes were differentially regulated. Apart from in the male spleen, more genes were differentially regulated upon rapamycin treatment, with 199/210 DEGs in kidney, 828/534 DEGs in spleen and 743/317 DEGs in muscle of female mice and male mice, respectively. The strongest changes occurred with combination treatment, with 290/105 DEGs in kidney, 4,038/2,391 DEGs in spleen and 967/479 DEGs in muscle of female mice and male mice, respectively. Gene Ontology (GO) enrichment analysis showed little overlap in the transcriptional responses in male and female mice (Fig. 7g–l). Only GO terms associated with chromosome segregation, nuclear division and microtubule cytoskeleton organization involved in mitosis were downregulated in the spleen of both male and female combination-treated animals (Fig. 7j). There was also little overlap in DEGs between rapamycin and trametinib (Fig. 7a–f), with GO showing different terms between rapamycin and trametinib single treatments (Fig. 7g–l). Although there was significant overlap in DEGs between the single and the combined drug treatments (Fig. 7a–f), most DEGs were specific to the combined treatment, and there was little overlap in GO terms between single and combined treatments (Fig. 7g–l), suggesting that the stronger effect in the combined treatment was not simply a combination of the individual drug effects. Many GO terms were associated with immune regulation and inflammation (Fig. 7g–l) in the kidney, both among the upregulated and downregulated genes (Fig. 7g,h). In contrast, in the spleen and muscle, genes associated with immune regulation and inflammation were exclusively downregulated (Fig. 7i–l), suggesting reduced inflammaging.

There was differential regulation of genes associated with inflammation. CD5 antigen-like (*Cd5l*) and C-C motif chemokine ligand 8 (*Ccl8*, also known as *Mcp2*) were strongly downregulated by combined treatment in female and male kidney and in male and female spleen and male muscle (Extended Data Fig. 7a–f). Increased levels of *Cd5l* and *Ccl8* are linked to inflammatory conditions^35^ and invasiveness and metastasis in various cancers^36^. *Cd5l* and *Ccl8* were also downregulated by rapamycin but not trametinib in the spleen (Extended Data Fig. 7b,e) and in tendency also in kidney (Extended Data Fig. 7a,d). Reduced expression of *Cd5l*, which is expressed mostly by macrophages in inflamed tissues^35^, suggests reduced macrophage infiltration of kidney tissue of combination-treated and rapamycin-treated animals. Lysozyme 1 (*Lyz1*) expression was strongly downregulated by combination treatment in both kidney and spleen of male and female mice (Extended Data Fig. 7g,h) but not in muscle. *Lyz1* is pro-inflammatory and can promote kidney fibrosis^37^. In kidney, *Lyz1* was downregulated by trametinib but not rapamycin (Extended Data Fig. 7g). In female but not male spleen, *Lyz1* was significantly downregulated by rapamycin and trametinib. Reduced *Lyz1* levels upon trametinib treatment suggest a protective role against kidney fibrosis, even more pronounced after co-administration of rapamycin. Combined drug treatment also reduced expression of chemokines and chemoattractants in the kidney, spleen and muscle, including the chemokine (C-X-C motif) ligand (Cxcl) genes *Cxcl7*, *Cxcl11* and *Cxcl13* (Extended Data Fig. 7i–l). *Cxcl7* was downregulated by rapamycin in male muscle and by the combined treatment in male muscle and kidney (Extended Data Fig. 7i,j). *Cxcl11* was significantly downregulated by rapamycin and the combination treatment in female spleen (Extended Data Fig. 7k). *Cxcl13* was in the top 10 of significantly downregulated genes by rapamycin in muscle, with a similar trend for the combination treatment (Extended Data Fig. 7l). *Cxcl14* was significantly upregulated by rapamycin and the combination treatment in muscles of both sexes (Extended Data Fig. 7m). *Cxcl14* was significantly downregulated in the female but not male spleen under combination treatment (Extended Data Fig. 7n). *Cxcl14* has context-dependent pro-tumor and anti-tumor effects^38^, possibly explaining the tissue-specific regulation. *Cxcl7*, *Cxcl11* and *Cxcl13* activate neutrophils and macrophages, promoting inflammatory states^39^, while also facilitating tumor growth, invasion and migration^40^. Their reduced expression upon combined drug treatment accords with the anti-inflammatory and anti-cancer effect of rapamycin and trametinib co-administration.

E26 avian leukemia oncogene 2, 3′ domain (Ets) transcription factors are key downstream effectors of the Ras/MEK/Erk signaling, and activated ETS2 sustains inflammatory responses by regulating the expression of inflammatory genes, including chemokines and matrix metallopeptidases (Mmp)^41^. Spleen tyrosine kinase (*Syk*), a direct target of ETS2 (ref. ^42^), was significantly downregulated by combination but not single-drug treatment in female kidney (Extended Data Fig. 8a). *Syk* was also downregulated by rapamycin and the combination treatment in male muscle (Extended Data Fig. 8b). *Syk* deficiency is protective against inflammation^41^, suggesting that its downregulation may have contributed to reduced inflammation in combination-treated mice. Expression of the two matrix metallopeptidases *Mmp2* and *Mmp13* was significantly downregulated in female but not male spleen upon combination treatment and with rapamycin for *Mmp2* in female spleen (Extended Data Fig. 8c,d). MMPs are involved in inflammation, and increased *Mmp13* and *Mmp2* expression is associated with increased tumor invasion and cancer progression^43^. *Mmp2* interacts with the α-2-macroglobulin (*A2m*) gene, which was among the strongest downregulated genes by rapamycin and combination treatment in spleen (Extended Data Fig. 8e). *A2m* modulates immune and inflammatory processes and promotes pro-proliferative and anti-apoptotic responses in cancer cells and macrophages^44^. *A2m* expression was also strongly downregulated by rapamycin and the combination treatment in the female kidney (Extended Data Fig. 8f). Elevated *A2m* levels are associated with kidney pathologies, including fibrosis and age-related kidney dysfunction^45^. Thus, rapamycin and the combination treatment may have alleviated age-related kidney fibrosis and dysfunction by lowering *A2m* levels. Interestingly, levels of *Mmp12* were strongly increased by rapamycin and the combination treatment in both the female and male spleen (Extended Data Fig. 8g). *Mmp12* usually promotes inflammation but has also been associated with anti-inflammatory effects^46^. Inflammatory gene expression was often sex specific. Although the drug treatments reduced Mmp expression only ín female mice, levels of chymotrypsin-like elastase (Cela) genes were exclusively downregulated in male mice (Extended Data Fig. 8h–j). *Cela2a* and *Cela3b* levels were significantly downregulated by single and combination drug treatments in male but not female spleen (Extended Data Fig. 8h,i). Furthermore, *Cela1* was downregulated by rapamycin in the male muscle (Extended Data Fig. 8j). Cela genes are associated with inflammatory states^47^ and are also upregulated in cancer^48^.

In summary, the treatment with rapamycin and trametinib induced a drug-specific, sex-specific and tissue-specific remodeling of the aging transcriptome characterized by reduced expression of genes associated with inflammation and cancer growth.

## Discussion

We established that the FDA-approved drug trametinib, an inhibitor of RAS/Mek/Erk signaling, is geroprotective in mice and that its combination with the mTOR inhibitor rapamycin has stronger effects than either drug alone in both female and male mice. The combination treatment produced an additive increase in lifespan, attenuated the decline in heart function with age, delayed tumor growth and overall tumor load and reduced brain and peripheral inflammation, suggesting improved health at old age. Several ongoing clinical trials address the potential of mTOR inhibitors as geroprotective drugs in humans^49^. Our study suggests that simultaneous inhibition of the mTOR and Ras–Mek–ERK pathway provides additional benefits that are worth exploring in humans.

Trametinib is used to treat metastatic melanomas and has also been used in clinical trials for treatment of solid tumors^50^. At a dose of 2 mg of trametinib per day, the drug is reasonably well tolerated, with side effects including skin rash, diarrhea, fatigue and retinopathy^50^. Average peak plasma levels in patients receiving 2 mg of trametinib daily were between 5.5 ng ml^−1^ and 7.5 ng ml^−1^ (ref. ^50^), which is significantly higher than the 0.1 ng ml^−1^ in mice fed with 1.44 mg of trametinib per kg of food. In a mouse model of mucosal melanoma, administering trametinib via oral gavage resulted in an average peak concentration of 23.8 ng ml^−1^ (ref. ^51^), which is more than 200-fold the concentration required for lifespan extension. Although effects of trametinib plasma levels might not be directly similar between chronic and acute application, these results suggest that higher trametinib doses are required for its anti-cancer effect than for lifespan extension. Consistently, tumor progression or load was not significantly changed in long-lived mice treated with trametinib, and the effects of 1.44 mg kg^−1^ trametinib on lifespan and health were mild. We, therefore, probably underdosed, and the optimal dose for geroprotection may be higher. In the pre-test, a dose of 2.88 mg kg^−1^ trametinib did not cause detrimental side effects and should be tested in future studies. It will also be important to assess the effects of late-onset and intermittent treatment, as these would be key to feasibility of and adherence to trametinib treatment in humans.

Combined treatment with rapamycin and trametinib increased lifespan by 29% and 27% in female and male mice, respectively, with no indication of any positive or negative interaction between the drugs, which had an additive effect on lifespan. It is not known what doses of rapamycin or trametinib produce a maximum increase in lifespan in mice or if these or their combination would also maximize late-life health. Nor is it known if the stronger geroprotection from combination treatment is a result of stronger target inhibition, additive inhibtion of more targets or some form of interaction between the drugs, for instance by prevention of signaling feedback.

Rapamycin caused liver lipidosis, hyperglycemia and testicular degeneration, whereas trametinib induced no obvious detrimental side effects. However, further in vivo experiments, including measurements of circulating insulin levels and glucose and insulin tolerance, will be necessary to confirm this. Apart from their longer lifespan, trametinib-treated mice showed only mild phenotypes, and the mechanisms contributing to their increased lifespan are currently unclear. Trametinib reduced spleen size at old age and caused a strong transcriptional response, especially in the male spleen, with an enrichment of GO terms associated with cell division and myeloid cell development. The Ras–Mek–Erk pathway controls B and T cell development, and Erk controls hyperproliferation in a mouse model of lymphoproliferative disease characterized by an enlargement of peripheral lymphoid organs^52^. Thus, trametinib may affect immunosenescence in the aging spleen.

Combination treatment with rapamycin and trametinib blocked the age-related increase in brain glucose uptake, and it also reduced density of activated microglia and astrocytes in several brain regions of female mice, indicative of reduced brain inflammation. Because trametinib did not cross the blood–brain barrier at the dose used, something else may have done so to decrease age-related inflammation in the brain. Treatment with the pro-longevity drugs acarbose, 17-α-estradiol and nordihydroguaiaretic acid specifically reduced age-associated inflammation in the hypothalamus of male but not female mice, whereas caloric restriction reduced brain inflammation in both sexes^53^. Geroprotection by drugs can be sex specific, and microglia show sex-related differences during development and aging^54^, so it will be important to assess the effects of combination treatment on brain inflammation in male mice. Another limitation of our study was that we could not measure the effect of rapamycin on brain glucose uptake and, therefore, did not address whether the effects of the combination treatment were caused by rapamycin. In addition, differences in region-specific glucose uptake and brain inflammation implicate additional currently unknown mechanisms.

The effects of the drugs were sex specific, especially at the molecular level. These included downregulation of plasma cytokines, which was more prominent in female mice, and the tissue-specific response in the transcriptome of aged animals. Sex-specific effects are commonly observed in longevity interventions^55^ and in interventions targeting the insulin–mTOR network^56^. Explanations could include differences in the aging process between male and female mice or in the bioavailability of the drugs. Indeed, trametinib plasma levels were significantly higher in female mice than in male mice. Rapamycin blood levels were also higher in female UM-Het3 mice^20^, but there was no sex difference in rapamycin plasma levels of the intermittently fed C3B6F1 mice used in this study^22^. Notably, despite higher plasma levels in female animals, the transcriptional response to trametinib treatment in the spleen was much stronger in male mice, suggesting that other processes than just drug bioavailability contribute to the sexually dimorphic molecular responses.

In summary, our results establish trametinib as a geroprotector that extends lifespan in mice. In combination with rapamycin, it acts additively to extend lifespan, and the drug combination induces late-life improvement in health at doses that have no detectable side effects. Trametinib should, therefore, be included in future trials to address whether it can geroprotect in humans, especially in combination with rapamycin.

## Methods

### Mouse husbandry and drug treatments

All animal protocols were done in accordance with the guidelines of the Federation of the European Laboratory Animal Science Association (FELASA) and approved by the Landesamt für Natur, Umwelt und Verbraucherschutz, Nordrhein-Westfalen, Germany (reference nos. 84-02.04.2017.A074 and 81-02.04.2019.A313).

Mice were housed in individually ventilated cages (Techniplast, GM500 Mouse IVC Green Line) in groups of five per cage, under specific pathogen-free conditions at 21 °C with 50–60% humidity and a 12-hour light/dark cycle. Mice had constant access to nesting material and chew sticks and ad libitum access to a standard rodent diet (Ssniff, R/MH low phytoestrogen; Ssniff Spezialdiäten GmbH) and sterile-filtered water. All mice were C3B6F1 hybrid, generated in-house by crossing C3H/HeOuJ female mice with C57BL/6NCrl male mice (strain codes 626 and 027, respectively; Charles River Laboratories).

For the trametinib pre-test experiment, 3-month-old male and female mice, five per treatment group, were fed with standard chow containing 0, 0.29, 0.58, 1.44, 2.88 or 11.52 mg of trametinib per kg of diet for 4 weeks. Mice were euthanized, and tissues were dissected for molecular and pathological analyses. At weaning, mice were fed a standard chow diet supplemented with the rapamycin encapsulation material Eudragit S100 (Evonik Cyro, 1-207-490-4242; 480 mg kg^−1^ diet). All experimental drug treatments started at 6 months of age, when control mice received a standard diet supplemented with the corresponding amount of Eudragit S100 (480 mg kg^−1^) and PEG-400 (3.2 ml kg^−1^). Animals treated with rapamycin received standard chow containing 42 mg kg^−1^ rapamycin (522 mg encapsulated rapamycin per kg) and PEG-400 (3.2 ml kg^−1^), and animals treated with trametinib received food containing 1.44 mg kg^−1^ trametinib, Eudragit S100 (480 mg kg^−1^) and PEG-400 (3.2 ml kg^−1^). Animals receiving the combined diet were fed with food containing 42 mg kg^−1^ rapamycin, 1.44 mg kg^−1^ trametinib and PEG-400 (3.2 ml kg^−1^). Trametinib was administered continuously. Rapamycin, whether alone or with rapamycin, was administered alternating between rapamycin and control food on a weekly basis^22^. Rapamycin was obtained from LC Laboratories and encapsulated by the Southwest Research Institute (SwRI). Trametinib was obtained from BIOZOL (1187431-43-1).

### Survival analysis

Survival of animals was assessed for a total of 876 mice: 482 male mice and 394 female mice. Mice for phenotyping, tissue collection and survival analysis were bred in three generations from the same breeding pairs. Mice of the phenotyping and tissue collection cohorts were included in the survival analysis and were censored at 24 months of age, when their tissues were collected. Health status of all mice was assessed daily using a standardized health score. Animals with poor health status were closely monitored and euthanized if they reached a predefined score. Mice were otherwise left undisturbed until they died of natural cause. Kaplan–Meier survival curves were generated using birth and death dates. Median lifespan was assessed, and survivorship was analyzed using log-rank test and Cox proportional hazard analysis. Maximum lifespan was assessed using the Wang–Allison test. Survival curves for control and rapamycin-treated mice of the lifespan cohort were previously published^22^.

### Tissue collection

Mice were euthanized using cervical dislocation. Tissues were dissected and snap frozen in liquid nitrogen. Plasma was isolated by adding 1 μl of 500 mM EDTA per 100 μl of blood and subsequent centrifugation at 1,000*g* for 10 minutes at 4 °C. Tissues for histopathological analysis were fixed in 10% neutral buffered formalin for preparation of formalin-fixed, paraffin embedded (FFPE) tissues.

### Cross-sectional histopathology

Cross-sectional histopathology of liver, heart, pancreas, kidney, WAT, brown adipose tissue (BAT), spleen and testes of 24-month-old animals was carried out by Robert Klopfleisch (Institute of Veterinary Pathology, Freie Universität Berlin). Hematoxylin and eosin stainings were scored. Liver tissue was examined for the presence of lymphomas, sarcomas, benign or malignant tumors, lipidosis (diffuse or focal) and necrosis. Liver tumor grade was determined as 1 (low), 2 (moderate), 3 (high) and 4 (very high). Heart tissue was assessed for hypertrophy and fibrosis. In pancreas, inflammation, fat necrosis, lymphoma and exocrine atrophy were evaluated. For heart and pancreas, pathology presence was scored as 1 and absence as 0. In kidney, lymphoma, oxalate crystals and hydronephrosis were documented, and kidney inflammation and glomerulopathy were scored with 1 (mild), 2 (moderate) and 3 (severe). WAT and BAT were scored for lymphoma, sarcoma, necrosis and granuloma and inflammation as 1 and absence of pathology as 0. In spleen, extramedullary hematopoiesis, lymphomas, sarcomas, germinal center and mantle zone hyperplasia were scored with 1 or 0 (absence). Testes were examined for atrophy or degeneration.

### Postmortem macropathology

Dead mice were assessed for tumor load and location, organ enlargement and abnormalities, including organ discoloration or granulation. The presence and severity of tumors was scored with 0 (absence), 1 (one tumor in one organ), 2 (multiple tumors or tumor diameter > 3 cm in one organ or metastasis in two organs) and 3 (severe metastasis in three or more organs). Other lesions were scored with 1 or 0 (absence). The severity of pathology was adjusted upon combination of multiple pathological findings.

### Liquid chromatography–mass spectrometry analysis of trametinib plasma levels

Next, 20 µl of plasma was mixed with 1 ml of a −20 °C methyl-tert-butyl-ether (MTBE):methanol:water (5:3:2 (v:v:v)) mixture, containing 10 ng ml^−1^ everolimus as internal standard. Samples were incubated for 30 minutes at 4 °C and then centrifuged at 21,000*g* and 4 °C for 10 minutes. Supernatant was transferred to fresh tubes, and 150 μl of MTBE and 100 µl of UPC/MS-grade water (BioSolve) were added and incubated for 15 minutes at 15 °C. Samples were centrifuged for 5 minutes, 16,000*g* at 15 °C. The MTBE phase was dried in a Speed Vacuum concentrator and resuspended in 80 µl of 70:30 (v:v) acetonitrile:isopropanol (BioSolve) containing 5% dimethylsulfoxid. Samples were vortexed, incubated for 5 minutes in a sonication bath, filtered through a 0.2-µm filter and then transferred to autosampler vials, and liquid chromatography–mass spectrometry (LC-MS) was performed using a Xevo TQ-S triple quadrupole mass spectrometer (Waters) coupled to an Acquity iClass liquid chromatography system (Waters). Samples were analyzed using a multiple reaction monitoring (MRM) method. For the absolute quantification of trametinib levels, trametinib standards were diluted to concentrations of 0, 2, 4, 10, 40, 200, 600, 1,200, 2,400 and 3,600 ng ml^−1^. LC–MS data were analyzed using TargetLynx software (version 4.1, Waters).

### Measurement of AST and ALP levels

Plasma measurements of AST and ALP levels were performed by Laboklin GmbH & Co. KG, Diagnostic Laboratory.

### Measurement of plasma glucose levels

Plasma glucose levels were measured using a glucose monitor (Roche, Accu-Chek Instant).

### Plasma proteomics (Olink)

Plasma protein levels were measured via proximity extension assay in EDTA plasma samples using the Olink Target 96 Mouse Exploratory panel (Olink Proteomics). Protein expression values were normalized using the Ct values of extension controls and a relative correction factor determined by Olink. Normalized protein expression (NPX) values on a log_2_ scale were analyzed. Eighty-four out of 86 plasma samples provided high-quality data. A total of 74 proteins were evaluated, representing greater than 75% quantification values as determined by values above the limit of detection. Individual protein measurements at old age between treatment groups were compared using Kruskal–Wallis and post hoc Mann–Whitney *U*-test with Benjamini–Hochberg correction (scipy Python library version 1.6.2).

### Protein extraction for western blot

Next, 5–10 mg of liver tissue was lysed in 400 μl of RIPA buffer (Pierce) containing cOmplete Mini Protease Inhibitor without EDTA (Roche) and PhosStop phosphatase inhibitors (Roche) in FastPrep Lysing Matrix D tubes (MP Biomedicals, 116913100) using FastPrep-24 (Thermo Fisher Scientific). Protein extracts were incubated on ice for 10 minutes and placed in a sonicator water bath filled for 10 minutes. Centrifugation for 15 minutes at 15,871*g* and 4 °C followed. Protein quantification was performed using a BCA Protein Assay Kit (Pierce, 23225). Samples were boiled; Laemmli buffer at ¼ of the total volume and 5% β-mercaptoethanol were added; and protein extracts were stored at −80 °C.

### Western blotting

Next, 20 μg of protein extracts was loaded and separated using 12% acrylamide gels (Criterion TGX Stain-Free Protein Gel; Bio-Rad, 5678044) and blotted on PVDF membranes (Amersham Hybond, GE10600023; Merck) at 80 V, for 1 hour, on ice. Membranes were blocked using 5% non-fat dry milk powder in Tris-buffered saline (TBS) 0.1% Tween 20 (TBS-T) at room temperature (RT) for 1 hour and then incubated with primary antibodies for phosphorylated ERK1/2 (1:1,000, anti-rabbit; Cell Signaling Technology, 4370) and total ERK1/2 (1:1,000, anti-rabbit; Cell Signaling Technology, 4695) in 5% fatty-acid-free BSA in TBS-T at 4 °C overnight. Blots were washed 3 × 15 minutes in TBS-T and incubated with HRP-coupled secondary antibodies (Thermo Fisher Scientific) diluted in 5% milk in TBS-T for 1 hour at RT, followed by 3 × 15-minute washes in TBS-T at RT. For signal development, ECL Select Western Blotting Detection Reagent (GE Healthcare) was applied, and image acquisition was performed using a ChemiDoc XRS+ System (Bio-Rad). Western blot signals were quantified using Image Lab Software (Bio-Rad) and normalized against non-drug-treated controls.

### Immunostaining of brains

Brains of 6-month-old control female mice and 24-month-old control, rapamycin, trametinib and combined female mice were dissected and fixed in 10% neutral buffered formalin for 2–4 hours at RT and overnight at 4 °C. Brains were dehydrated in 30% sucrose in 1× PBS and frozen in Tissue-Tek OCT (Labtech) on dry ice. Then, 25-μm brain sections were cut using a cryostat (Leica Biosystems, CM1850) and mounted on Superfrost Plus microscope slides (Thermo Fisher Scientific). For immunostainings, slides were washed with PBS for 10 minutes, permeabilized with PBS + 0.2% Triton X-100 (PBST) for 15 minutes and washed in PBST for 5 minutes. Samples were incubated with 2% normal goat serum in PBST at RT and then incubated with primary antibodies in 2% normal goat serum in PBST overnight at 4 °C. Primary antibodies included: ionized calcium-binding adaptor molecule 1 (Iba-1, 1:500; Wako, 019-19741) and glial fibrillary acidic protein (GFAP, 1:1,000; Sigma-Aldrich, G3893). Slides were washed 3 × 15 min in PBST and incubated for 1 hour with Alexa Fluor-conjugated secondary antibodies at 1:1,000 in 2% normal goat serum at RT. Secondary antibodies included: Alexa Fluor 488 goat anti-rabbit IgG (Thermo Fisher Scientific, A-11008, to detect Iba-1) and Alexa Fluor 633 goat anti-mouse IgG (Thermo Fisher Scientific, A-21126, to detect GFAP). Slides were washed 2 × 10 min in PBST and 1 × 10 min in PBS and incubated for 30 min with DAPI (1:10,000 in PBS). Slides were washed with PBS for 10 min and embedded using VECTASHIELD Antifade Mounting Medium without DAPI (VectorLabs).

### Confocal imaging and quantification of astrocyte and microglia density

Imaging was performed using a Leica SP8 upright confocal microscope and Application Suite X 3.0.15 software (Leica Microsystems). Then, 40-μm *z*-stack confocal images were acquired at 2-μm intervals, with ×40/1.3 oil objective. Settings were standardized for all treatment groups during image acquisition. Representative montages from *z*-stack confocal images were used, and image processing was performed using ImageJ (Fiji) software version 2.3.0/1.53q. For quantification of astrocytes and microglia density, the cell counter tool was used to count GFAP^+^ or Iba-1^+^ cells that overlapped with DAPI^+^ nuclei, respectively, in 10–15 images per brain region per mouse brain.

### RNA isolation

Next, 4–10 mg of spleen, muscle and kidney samples were homogenized in 1 ml of TRIzol (Life Technologies) using FastPrep-24. Samples were incubated at RT for 5 minutes. Then, 200 μl of chloroform was added, and samples were incubated at RT for 10 minutes and centrifuged at 12,000 g for 15 minutes at 4 °C. The aqueous phase was transferred to a new RNAse-free tube. Then, 500 μl of isopropanol, 50 μl of 3.0 M NaOAc and 1.5 μl of glycogen (Thermo Fisher Scientific) were added, and the mix was incubated at −80 °C for 30 minutes. Samples were centrifuged at 12,000*g* for 10 minutes at 4 °C. RNA pellets were washed twice with 1 ml of ice-cold 70% ethanol, air dried for 5–10 minutes and resuspended in 30 μl of RNAse-free water. RNA samples were treated with DNAse using a TURBO DNAse Kit (Thermo Fisher Scientific).

### RNA-seq analysis

RNA-seq library preparation and sequencing of kidney, spleen and muscle RNA was performed by Admera Health. poly(A)^+^ mRNA was isolated using a NEBNext Poly(A) mRNA Magnetic Isolation Kit (New England Biolabs). Prior to first-strand synthesis, samples were randomly primed (5′ d(N6) 3′ (*N* = A,C,G,T)) and fragmented. First-strand synthesis was done using ProtoScript II Reverse Transcriptase. Library construction was done according to a NEBNext Ultra II Non-Directional RNA Library Prep Kit for Illumina (New England Biolabs, E7760L). Final libraries quantity was assessed by Qubit 2.0 (Thermo Fisher Scientific), and quality was assessed by TapeStation D1000 ScreenTape (Agilent Technologies). Illumina 8-nt dual indices were used. Equimolar pooling of libraries was performed based on QC values and sequenced on an Illumina NovaSeq S4 with a read length configuration of 150 bp for 50 million paired-end reads per sample. Libraries read processing was automated using Flaski (version 3.11.34). Raw reads were mapped to the mm39 ENSEMBL build 105 using kallisto (version 0.46.1). Gene counts were quantified in the same step. Differential gene expression was analyzed using DESeq2 (version 1.24.0) in R (version 3.6.3). Data were processed using numpy (version 1.12.0), scipy (version 1.7.1) and pandas (version 1.2.0). Results were visualized using matplotlib (version 3.3.2), matplotlib-venn (version 0.11.5) and seaborn (version 0.11.1). GO enrichment analysis was performed using the DAVID API-based function DAVIDenrich of the AGEpy Python package (version 0.8.2) on the GOTERM_BP_FAT ontology to identify significantly enriched terms (Benjamini–Hochberg-adjusted *P* < 0.05). As background, all expressed genes of a tissue were used. Redundancy of significantly enriched GO terms (adjusted *P* < 0.05) was reduced using REVIGO (cutoff: 0.7, valueType: pvalue, measure: SIMREL). GO terms were visualized using dot plot representation.

### ^18^F-FDG PET/CT Imaging

PET imaging was as described in ref. ^57^ using an Inveon preclinical PET/CT system (Siemens). CT data were used for attenuation correction of the PET data, and the CT image of the scull was used for image co-registration. Plasma glucose levels were determined from a tail vein blood sample using a standard glucometer (Bayer) after removing the tail vein catheters. PET data were histogrammed in timeframes of 12 × 30 s, 3 × 60 s, 3 × 120 s and 7 × 240 s, rebinned in three dimensions (3D), and images were reconstructed using the MAP-SP algorithm provided by the manufacturer. The imaging analysis software Vinci was used to co-register images to a 3D mouse brain atlas constructed from the two-dimensional (2D) mouse brain atlas (Paxinos). Kinetic modeling was performed as described in ref. ^57^. The ratio of tissue and plasma glucose concentrations (CE/CP) is a measure for glucose transport and is given by CE/CP = K1 / (k2 + k3 / 0.26)^26,57^. Because cellular activation is accompanied by increased glucose transport and this parameter is less sensitive to changes in plasma glucose level, we use alterations of glucose transport (CE/CP) as surrogate for alterations in neuronal activation. Statistical testing was performed by application of a voxel-wise *t*-test between groups. 3D maps of *P* values allow for identification of regions with significant differences in the parameters. From these regions, we defined volumes of interest (VOIs) and performed additional statistical testing.

### Mouse phenotyping

A separate mouse cohort of 15 mice per treatment group was used to evaluate drug-related and age-related changes in healthspan, behavior and fitness. Experiments were performed at 12 months (middle-aged) and 20–22 months (old) of age. All tests were carried out during the light phase, and experimenters were blinded to the treatment group.

### Electrocardiography

Electrocardiography (ECG) was performed non-invasively using the ECGenie system (Mouse Specifics). For acclimatization, each mouse was placed on the training platform for 10 minutes and then placed on the measurement platform on interchangeable electrodes that transmit the electrical signals to a computer for a maximum of 10 minutes. Signals with a heart rate variation that exceeded 35 bpm were excluded, and average heart rate per mouse was evaluated.

### Rotarod

Motor coordination was assessed using a Rotarod (TSE Systems, type 3375-M5). Measurement started at low speed (5 r.p.m.), and the revolving rate was continuously increased to 40 r.p.m. over a period of 300 s. The total time that each mouse spent on the rod, with a cutoff time of 300 s, was measured. The test was performed on four consecutive days, and only data from day four were used for the analysis.

### Treadmill

Endurance was measured using a treadmill (TSE Systems, type 3033401-M-04/C). Mice were acclimatized to the experimental setup for 5 minutes under low speed (0.1 m s^−1^). A progressively accelerating speed was applied from 0.1 m s^−1^ for another 10 minutes to 1.3 m s^−1^ within 60 minutes. A mild electroshock (0.3 mA) was applied for 5 s, followed by 5 s of refractory period as soon as mice slowed down beyond a critical point and crossed the laser beam for more than 2 s to ensure that mice stopped running only upon exhaustion. Exhaustion was defined as the willingness of a mouse to withstand three consecutive shocks instead of continuing running.

### Open field

For the open field experiment using a ActiMot2 (TSE Systems), mice were placed in a 50 cm × 50 cm × 40 cm box and were allowed to freely explore for 10 minutes. The test chamber was illuminated to 20–30 lux. Total activity, speed and time spent in the center were recorded via infrared sensors.

### Y-maze

Mice were placed in a Y-shaped maze and their activity recorded for 5 minutes using a video tracking system (TSE Systems, VideoMot). Global speed indicates the average speed of the mice during the 5-minute measurement period. Percent alternating arm visits were calculated as a read-out for spatial working memory.

### Statistics and reproducibility

Experiments were performed in a randomized and blinded fashion whenever possible. For the survival, phenotyping and tissue collection cohorts, female mice were randomly allocated to cages after weaning using simple randomization. Male mice were weaned litter-wise to reduce aggressive behavior. If male mice of different litters had to be combined, a ratio of 2:3 was preferred over 4:1. Cages were assigned to treatment groups using simple randomization. Tissue samples used for histopathology, RNA-seq, plasma protein and trametinib level measurements were randomized prior to extraction/analysis using simple randomization. Mouse survival data and postmortem pathology were scored by mouse caretakers, who were unaware of the study design. All phenotyping experiments were performed with the experimenters blinded to treatment. Histopathology was scored in a blinded manner by an external pathologist. Plasma protein levels and RNA-seq measurements were performed by external companies in a blinded manner. Confocal imaging and image analysis were performed in a blinded manner. ^18^-FDG-CT/PET scans were measured by an external facility with the experimenters unaware of study design. In ECG measurements, signals with a heart rate variation that exceeded 35 bpm were excluded. Three samples were excluded from the RNA-seq analysis (1× male muscle trametinib, 1× female muscle combined and 1× female kidney rapamycin), as they presented as clear outliers in the principal component analysis (PCA). Otherwise, no data were excluded from the analyses. No statistical methods were used to pre-determine sample sizes, but our sample sizes are similar to those reported in previous publications^10,22^. Data distribution was assumed to be normal, but this was not formally tested. Statistical analysis was performed using GraphPad Prism 9.0 and 10.3.0, except for Cox proportional hazard analysis, which was performed in R. Number of animals and statistical tests are indicated in the figures and figure legends. For multiple comparison testing, one-way and two-way ANOVA were followed by Bonferroni post hoc test. To test for differences in the proportion of mice affected by pathologies, chi-square test and Poisson regressions were used. The counts of mice where the pathological finding was present or absent was set as the dependent variable, and the absence of pathology (score 0) was set as the reference classification. Comparisons were performed against control mice as reference.

### Reporting summary

Further information on research design is available in the Nature Portfolio Reporting Summary linked to this article.

## Supplementary information

Reporting Summary

Supplementary Table 1 Raw data of plasma proteins measured via the Olink Target 96 Mouse Exploratory panel in 24-month-old male and female mice.

Supplementary Table 2 Significantly regulated genes based on the RNA-seq analysis in 24-month-old mice treated with rapamycin, trametinib and the combination.

## Source data

Source Data Fig. 1 Unprocessed western blot images corresponding to Fig. 1d,e.

Source Data Fig. 2 Unprocessed western blot images corresponding to Extended Data Fig. 1a–c.

## Figures and Tables

**Extended Data Fig. 2:**
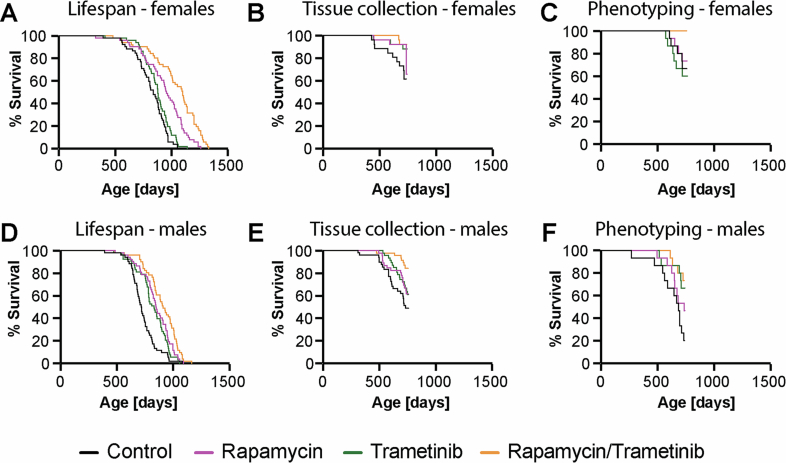
Survival data of animals from the lifespan, tissue collection and phenotyping cohorts. Survival curves of **a**–**c** female and **d**–**f** male mice of the **a**, **d** lifespan, **b**, **e** tissue collection and **c**, **f** phenotyping cohort. The corresponding pooled data are presented in Fig. 2. (Sample size lifespan cohort: control ♂ n = 52, ♀ n = 52, rapamycin ♂ n = 52, ♀ n = 51, trametinib ♂ n = 52, ♀ n = 51, combined ♂ n = 51, ♀ n = 51; tissue collection cohort: control ♂ n = 53, ♀ n = 33, rapamycin ♂ n = 53, ♀ n = 32, trametinib ♂ n = 54, ♀ n = 32, combined ♂ n = 54, ♀ n = 32; phenotyping cohort n = 15 for all treatments). Trametinib significantly extended lifespan in male mice and the combined treatment significantly increased lifespan compared to the individual drug treatments in both males and females when only survival of the lifespan cohort was analyzed. In the female lifespan cohort, trametinib showed a similar trend but the effect on lifespan was not significant, due to the limited power of the smaller sample size (females lifespan cohort: control vs rapa p < 0,0001, control vs tram p < 0.0697, control vs rapa/tram p < 0,0001, rapa vs tram p = 0.0005, rapa vs rapa/tram p = 0.0004, tram vs rapa/tram p < 0.0001; males lifespan cohort: control vs rapa p < 0,0001, control vs tram p = 0,0020, control vs rapa/tram p < 0,0001, rapa vs tram p = 0.1799, rapa vs rapa/tram p = 0.0283, tram vs rapa/tram p = 0.0006, log rank test). Survival curves for control and rapamycin treated mice of the lifespan cohort were previously published^22^. Within the different cohorts all treatments were run in parallel.

## Data Availability

Raw and processed RNA-seq data are available in the Gene Expression Omnibus under accession number GSE288795. Raw data for the Olink plasma proteome analysis are provided in Supplementary Table 1. Source data files for western blot analyses are available in the Supplementary Information files. All other data are available from the corresponding authors upon reasonable request. Source data are provided with this paper.
