# Supplementary material for: The geroprotectors trametinib and rapamycin combine additively to extend mouse healthspan and lifespan
Source: Nat Aging. 2025 May 28;5(7):1249–65. doi: 10.1038/s43587-025-00876-4 (PMC12270913; doi:10.1038/s43587-025-00876-4)
Supplement: Supplementary file 1 — Reporting Summary [file 43587_2025_876_MOESM1_ESM.pdf]

nature portfolio

Corresponding author(s): Sebastian Grönke and Linda Partridge

Last updated by author(s): Apr 11, 2025

Reporting Summary

Nature Portfolio wishes to improve the reproducibility of the work that we publish. This form provides structure for consistency and transparency in reporting. For further information on Nature Portfolio policies, see our Editorial Policies and the Editorial Policy Checklist.

Statistics

For all statistical analyses, confirm that the following items are present in the figure legend, table legend, main text, or Methods section.

n/a	Confirmed	
☐	☒	The exact sample size (*n*) for each experimental group/condition, given as a discrete number and unit of measurement
☐	☒	A statement on whether measurements were taken from distinct samples or whether the same sample was measured repeatedly
☐	☒	The statistical test(s) used AND whether they are one- or two-sided<br>*Only common tests should be described solely by name; describe more complex techniques in the Methods section.*
☐	☒	A description of all covariates tested
☐	☒	A description of any assumptions or corrections, such as tests of normality and adjustment for multiple comparisons
☐	☒	A full description of the statistical parameters including central tendency (e.g. means) or other basic estimates (e.g. regression coefficient) AND variation (e.g. standard deviation) or associated estimates of uncertainty (e.g. confidence intervals)
☐	☒	For null hypothesis testing, the test statistic (e.g. *F*, *t*, *r*) with confidence intervals, effect sizes, degrees of freedom and *P* value noted<br>*Give P values as exact values whenever suitable.*
☒	☐	For Bayesian analysis, information on the choice of priors and Markov chain Monte Carlo settings
☐	☒	For hierarchical and complex designs, identification of the appropriate level for tests and full reporting of outcomes
☒	☐	Estimates of effect sizes (e.g. Cohen's *d*, Pearson's *r*), indicating how they were calculated

Our web collection on statistics for biologists contains articles on many of the points above.

Software and code

Policy information about availability of computer code

Data collection	Image acquisition: Leica Application Suite X version 3.0.15
Data analysis	LC-MS data were analysed using TargetLynx (Version 4.1, Waters).<br>The python package scipy (v1.6.2) was used to analyse plasma protein levels (Olink) using Kruskall-Wallis and post-hoc Mann Whitney U-test with Benjamini-Hochberg correction.<br>Western Blot signals were quantified using Image Lab™ (6.1.0.07, Bio-Rad).<br>Processing of confocal images was performed using Image J (Fiji) software v2.3.0/1.53q (Image J, Maryland, USA) and the integrated cell counter tool.<br>RNA Seq librariy read processing was automated using Flaski (v3.11.34). Raw reads were mapped to the mm39 ENSEMBL build 105 using kallisto (v0.46.1). Differential gene expression was analysed using DESeq2 (v1.24.0) in R (v 4.2.2). Data were processed using the following python packages: numpy (v 1.12.0), scipy (v 1.7.1), and pandas (v 1.2.0). Results were visualized using matplotlib (v 3.3.2), matplotlib-venn (v 0.11.5), and seaborn (v 0.11.1). Gene ontology enrichment analysis was performed using the DAVID API based function DAVIDenrich of the AGEpy python package (v 0.8.2).<br>Volume Imaging in Neurological Research (Vinci) was used to co-register images to a 3D mouse brain atlas in the 18F-FDG PET/CT analysis. PET images were reconstructed using the MAP-SP algorithm (Inveon preclinical PET/CT, Siemens). Statistical analyses was performed using GraphPad Prism 9.0 and 10.3.0. Cox Proportional Hazard analysis was performed in R (R Core Team).

For manuscripts utilizing custom algorithms or software that are central to the research but not yet described in published literature, software must be made available to editors and reviewers. We strongly encourage code deposition in a community repository (e.g. GitHub). See the Nature Portfolio guidelines for submitting code & software for further information.

Data

Policy information about availability of data

All manuscripts must include a data availability statement. This statement should provide the following information, where applicable:

- Accession codes, unique identifiers, or web links for publicly available datasets
- A description of any restrictions on data availability
- For clinical datasets or third party data, please ensure that the statement adheres to our policy

Raw and processed RNA seq data are available in Gene Expression Omnibus under accession number GSE288795. Raw data for the Olink plasma proteome analysis are provided in Supplementary Table 1. Source data files for Western blot analyses are available in the supplementary information files. All other data are available from the corresponding authors upon reasonable request.

Research involving human participants, their data, or biological material

Policy information about studies with human participants or human data. See also policy information about sex, gender (identity/presentation), and sexual orientation and race, ethnicity and racism.

Reporting on sex and gender	N/A
Reporting on race, ethnicity, or other socially relevant groupings	N/A
Population characteristics	N/A
Recruitment	N/A
Ethics oversight	N/A

Note that full information on the approval of the study protocol must also be provided in the manuscript.

Field-specific reporting

Please select the one below that is the best fit for your research. If you are not sure, read the appropriate sections before making your selection.

☒ Life sciences ☐ Behavioural & social sciences ☐ Ecological, evolutionary & environmental sciences

For a reference copy of the document with all sections, see nature.com/documents/nr-reporting-summary-flat.pdf

Life sciences study design

All studies must disclose on these points even when the disclosure is negative.

Sample size	Sample size for the survival analysis and phenotyping experiments was determined by power analysis using G*power (Wilcoxon-Mann-Whitney test ,α, 0.05, β 0.2. Cohen´s d 0.8).
Data exclusions	In ECG measurements, signals with a heart rate variation that exceeded 35 bpm were excluded, according to Thireau et al., 2008. Three samples were excluded from the RNA seq analysis (1x male muscle trametinib, 1x female muscle combined, 1x female kidney rapamycin), as they presented as clear outliers in the PCA analysis. Otherwise, no data were excluded from the analyses.
Replication	Due to time restrictions the mouse data were not replicated.
Randomization	For the survival, phenotyping and tissue collection cohorts, female mice were randomly allocated to cages after weaning using simple randomization. Male mice were weaned litter-wise to reduce aggressive behaviour. If male mice of different litters had to be combined, a ratio of 2:3 was preferred over 4:1. Cages were assigned to treatment groups using simple randomization. Tissue samples used for histopathology, RNA seq, plasma protein and trametinib level measurements were randomized prior to extraction/analysis using simple randomization.
Blinding	Experiments were performed in a blinded fashion whenever possible. Mouse survival data and post-mortem pathology were scored by mouse care takers, who were unaware of the study design. All phenotyping experiments were performed with the experimenters blinded to treatment. Histopathology was scored in a blinded manner by an external pathologist. Plasma protein levels and RNA seq measurements were performed by external companies in a blinded manner. Confocal imaging and image analysis were performed in a blinded manner. 18-FDG-CT/PET scans were measured by an external facility with the experimenters unaware of study design.

Reporting for specific materials, systems and methods

We require information from authors about some types of materials, experimental systems and methods used in many studies. Here, indicate whether each material, system or method listed is relevant to your study. If you are not sure if a list item applies to your research, read the appropriate section before selecting a response.

Materials & experimental systems

n/a	Involved in the study
☐	☒ Antibodies
☒	☐ Eukaryotic cell lines
☒	☐ Palaeontology and archaeology
☐	☒ Animals and other organisms
☒	☐ Clinical data
☒	☐ Dual use research of concern
☒	☐ Plants

Methods

n/a	Involved in the study
☒	☐ ChIP-seq
☒	☐ Flow cytometry
☒	☐ MRI-based neuroimaging

Antibodies

Antibodies used

Primary Antibodies:
Ionised calcium-binding adaptor molecule 1 (Iba-1) , (019-19741, Wako)
Glial fibrillary acidic protein (GFAP) (G3893, Sigma)
Phospho-p44/42 MAPK (Erk1/2) (Thr202/Tyr204) (D13.14.4E) XP (Cell Signaling Technology, 4370)
p44/42 MAPK (Erk1/2) (137F5) Rabbit mAb (Cell Signaling Technology, 4695)
α-Tubulin (11H10) (Cell Signaling Technology, 2125)

Secondary Antibodies:
Goat anti-Rabbit IgG (H+L) Cross-Adsorbed Secondary Antibody, Alexa Fluor 488 (ThermoFisher, A-11008)
Goat anti-Mouse IgG1 Cross-Adsorbed Secondary Antibody, Alexa Fluor 633 (ThermoFisher, A-21126)
Goat anti-Rabbit IgG (H+L) Secondary Antibody, HRP (ThermoFisher, 31460)

Validation

Iba-1(019-19741, Wako) cited by 491 (https://www.labome.com/product/Wako-Chemicals-USA/019-19741.html)

GFAP) (G3893, Sigma) cited by 1329 (https://www.sigmaaldrich.com/DE/en/search/g3893?focus=papers&page=1&perpage=30&sort=relevance&term=G3893&type=citation_search)

Phospho-p44/42 MAPK (Cell Signaling Technology, 4370) cited by 8716 (https://www.cellsignal.com/products/primary-antibodies/phospho-p44-42-mapk-erk1-2-thr202-tyr204-d13-14-4e-xp-rabbit-mab/4370)

p44/42 MAPK (Erk1/2) cited by 6689 (https://www.cellsignal.com/products/primary-antibodies/p44-42-mapk-erk1-2-137f5-rabbit-mab/4695)

αα-Tubulin (11H10) (Cell Signaling Technology, 2125) cited by 817 (https://www.cellsignal.com/products/primary-antibodies/a-tubulin-11h10-rabbit-mab/2125)

Alexa Fluor 488-goat anti-rabbit IgG (A-11008, ThermoFisher Scientific) cited by 9,725 (https://www.thermofisher.com/antibody/product/Goat-anti-Rabbit-IgG-H-L-Cross-Adsorbed-Secondary-Antibody-Polyclonal/A-11008)

Alexa Fluor 633-goat anti-mouse IgG (A-21126, ThermoFisher Scientific) cited by 103 (https://www.thermofisher.com/antibody/product/Goat-anti-Mouse-IgG1-Cross-Adsorbed-Secondary-Antibody-Polyclonal/A-21126)

Goat anti-Rabbit IgG HRP (31460, ThermoFisher) cited by 2836 (https://www.thermofisher.com/antibody/product/Goat-anti-Rabbit-IgG-H-L-Secondary-Antibody-Polyclonal/31460)

Animals and other research organisms

Policy information about studies involving animals; ARRIVE guidelines recommended for reporting animal research, and Sex and Gender in Research

Laboratory animals

Mus musculus, C3B6F1 hybrid mice were generated in-house by crossing C3H/HeOuJ females with C57BL/6NCrl males (strain codes 626 and 027, respectively, Charles River Laboratories). Lifespan and phenotyping animals were observed throughout their whole life. Animals used for the pre-test to establish trametinib concentrations were 4 months old. Phenotyping experiments were performed at 12 and 24 months of age, apart from indirect calorimetry, which was done at 18 months of age. PET/CT measurements were done on the same animals at 12, 18 and 24 months of age. Tissues for histopathology and molecular analyses were collected at 24 months of age.

Wild animals

The study did not involve wild animals.

Reporting on sex

Most measurements including lifespan, phenotyping, transcriptomic and plasma protein levels were done in both male and female mice and data are presented separately for each sex. 18-FDG PET/CT and brain inflammation measurements were only done in female animals due to limited capacity of the PET/CT facility and availability of brain tissue, respectively.

Field-collected samples	The study did not involve field collected samples.
Ethics oversight	The mouse study was approved by the Landesamt für Natur, Umwelt und Verbraucherschutz, Nordrhein-Westfalen, Germany (reference no. 84-02.04.2017.A074 and 81-02.04.2019.A313).

Note that full information on the approval of the study protocol must also be provided in the manuscript.

Plants

Seed stocks	N/A
Novel plant genotypes	N/A
Authentication	N/A
