# Supplementary figures and images for: The geroprotectors trametinib and rapamycin combine additively to extend mouse healthspan and lifespan
Source: Nat Aging. 2025 May 28;5(7):1249–65. doi: 10.1038/s43587-025-00876-4 (PMC12270913; doi:10.1038/s43587-025-00876-4)

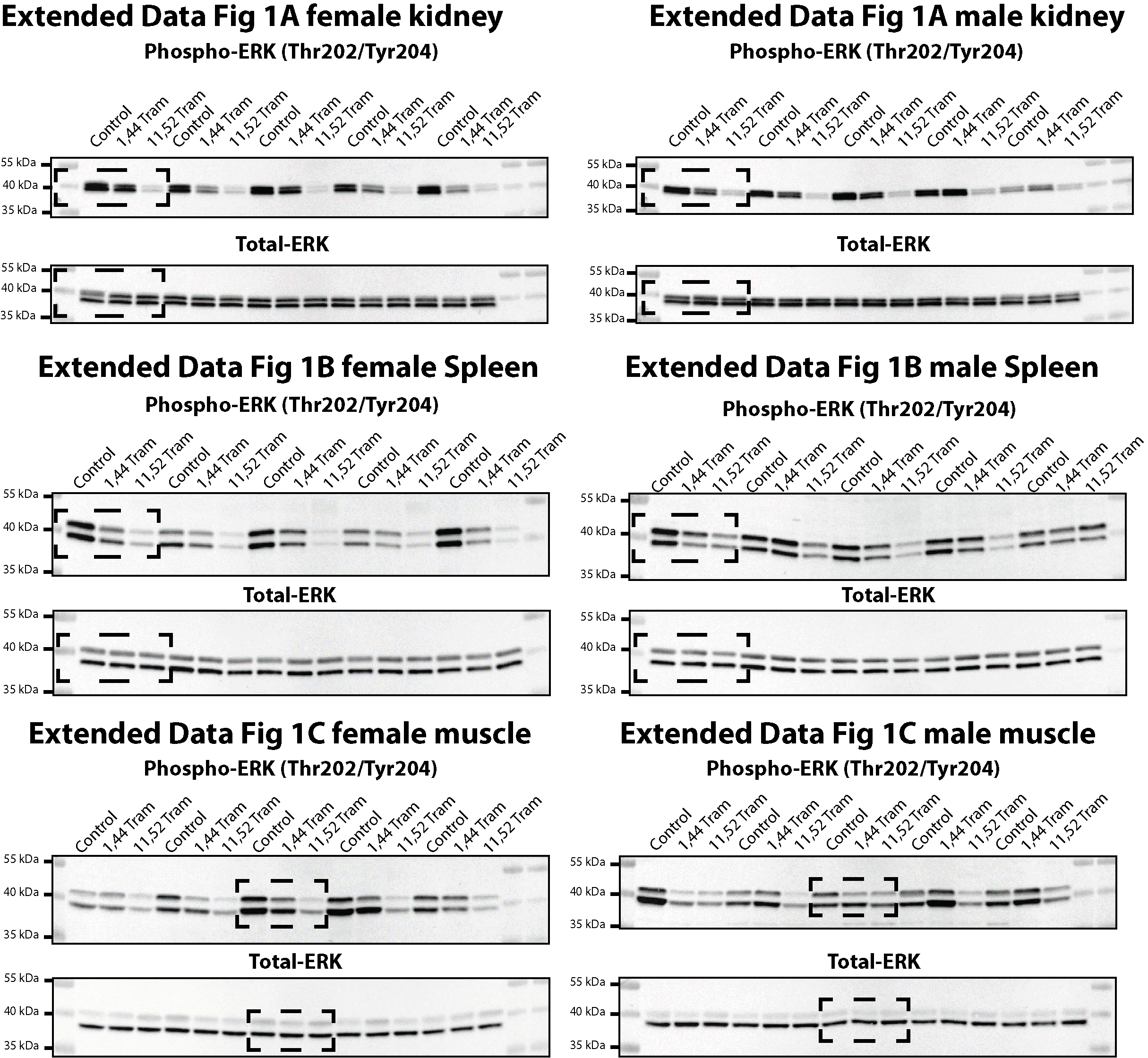

Supplement: Supplementary file 4 — Unprocessed western blot images corresponding to Fig. 1d,e. [file 43587_2025_876_MOESM4_ESM.tif]

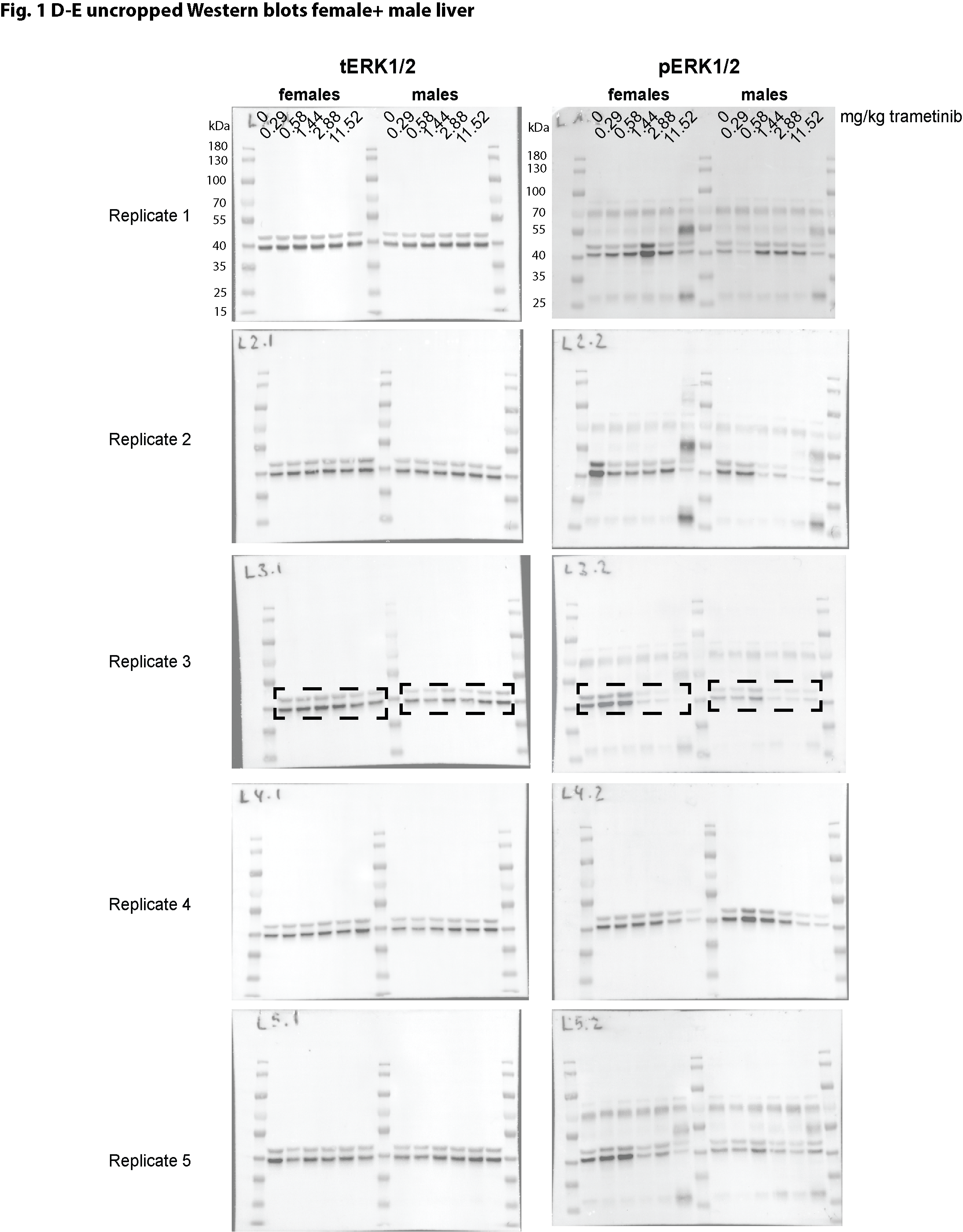

Supplement: Supplementary file 5 — Unprocessed western blot images corresponding to Extended Data Fig. 1a–c. [file 43587_2025_876_MOESM5_ESM.tif]
